# Varying Immunizations With *Plasmodium* Radiation-Attenuated Sporozoites Alter Tissue-Specific CD8^+^ T Cell Dynamics

**DOI:** 10.3389/fimmu.2018.01137

**Published:** 2018-05-28

**Authors:** Roland Frank, Michael Gabel, Kirsten Heiss, Ann-Kristin Mueller, Frederik Graw

**Affiliations:** ^1^Centre for Infectious Diseases, Parasitology Unit, University Hospital Heidelberg, Heidelberg, Germany; ^2^Centre for Modeling and Simulation in the Biosciences, BioQuant-Center, Heidelberg University, Heidelberg, Germany; ^3^German Center for Infection Research (DZIF), Heidelberg, Germany

**Keywords:** malaria, *Plasmodium berghei*, radiation-attenuated sporozoites, liver-resident CD8^+^ T cells, mathematical modeling, experimental vaccination

## Abstract

Whole sporozoite vaccines represent one of the most promising strategies to induce protection against malaria. However, the development of efficient vaccination protocols still remains a major challenge. To understand how the generation of immunity is affected by variations in vaccination dosage and frequency, we systematically analyzed intrasplenic and intrahepatic CD8^+^ T cell responses following varied immunizations of mice with radiation-attenuated sporozoites. By combining experimental data and mathematical modeling, our analysis indicates a reversing role of spleen and liver in the generation of protective liver-resident CD8^+^ T cells during priming and booster injections: While the spleen acts as a critical source compartment during priming, the increase in vaccine-induced hepatic T cell levels is likely due to local reactivation in the liver in response to subsequent booster injections. Higher dosing accelerates the efficient generation of liver-resident CD8^+^ T cells by especially affecting their local reactivation. In addition, we determine the differentiation and migration pathway from splenic precursors toward hepatic memory cells thereby presenting a mechanistic framework for the impact of various vaccination protocols on these dynamics. Thus, our work provides important insights into organ-specific CD8^+^ T cell dynamics and their role and interplay in the formation of protective immunity against malaria.

## Introduction

Despite recent advances and the regulatory approval of the RTS,S vaccine (1–4), the development of an efficient vaccine still remains an urgent priority and one of the major challenges in malaria research. Causing abrogation of parasite development during or shortly after the pre-pathological liver stage of malaria infection, either by radiation of sporozoites, their genetic modification or combined delivery with drug-treatment, has been proven to be the most successful approach to confer sterile protection against malaria infection (5–13). These whole sporozoite vaccination (WSV) strategies, of which, to date, radiation-attenuated sporozoites (RAS) are still the clinically most advanced approach, were shown to induce high levels of protective immunity in humans, non-human primates (NHP) and various mouse models (5, 11, 12). However, many aspects of these approaches still remain to be elucidated to allow the systematic design of effective vaccination protocols. This includes the identification of the actual immune responses mediating protection, as well as the processes regulating their generation and long-term maintenance (13, 14).

Especially studies in animal models, particularly in inbred mice, identified intrahepatic CD8^+^ memory T cells as the most crucial component mediating protective pre-erythrocytic immunity induced by RAS and other WSV approaches (15–19). The essential role of CD8^+^ T cells for protection was recently more precisely defined by the characterization of tissue-resident memory CD8^+^ T cells (T_RM_) within the liver. These cells shared a specific expression signature, which was largely consistent in two rodent models and correlated with protection in C57BL/6 mice immunized with *Plasmodium berghei* RAS (*Pb*RAS) (20, 21). Clinical WSV studies also suggested a critical role for liver-resident CD8^+^ T cell responses, mainly due to a paucity of blood-specific immune correlates of protection and supportive results from studies in NHP (11–13). The effective generation of such local immunity allowing for potent regional recall responses might, therefore, be of utmost importance for the rational design of protective vaccination strategies against malaria (21).

Despite these advances in our current knowledge on protective immune mechanisms and the availability of data from clinical trials, the implementation of WSV approaches still faces major challenges for wide-scale human use. These challenges comprise the development of effective vaccination schedules with regard to the dose and frequency of immunizations. Various regimens have been tested in experimental (17, 19, 22) and clinical trials (12, 13) demonstrating particularly for RAS a requirement for high doses and multiple numbers of intravenous (i.v.) injections. Due to the inaccessibility of the critical tissue-specific CD8^+^ T cell responses, especially in humans, it remains unclear how these cell populations are formed, maintained, and influenced by alterations in the vaccination regimens.

Several studies in mice have conducted longitudinal analyses of tissue-associated CD8^+^ T cell responses in spleen and liver following i.v. immunization with RAS. These studies reported (i) increasing frequencies of hepatic CD8^+^ T effector memory cells (T_EM_) after consecutive booster immunizations, (ii) exceedingly high threshold frequencies of antigen-specific CD8^+^ T cell responses necessary for protection, and (iii) different levels of protection dependent on the vaccination protocol (16, 17, 22–24). Despite these findings, we still lack a systematic and quantitative understanding of the differentiation, proliferation, interaction, and maintenance of specific CD8^+^ T cell subsets in response to vaccination.

To determine how exactly variations in the vaccine dose or number of injections affect these dynamics and, finally, the level and duration of protection, we systematically analyzed the effects of different *Pb*RAS vaccination regimens on the development of tissue-specific CD8^+^ T cell responses. To this end, we immunized groups of C57BL/6 mice by intravenous injection of *Pb*RAS varying the vaccine dose, the number of injections and the time to analysis. We then used mathematical modeling to infer the underlying dynamics and interactions of different CD8^+^ T cell subpopulations, with a focus on the central roles of the spleen and the liver in the formation of protective immunity.

Our data revealed differences in the cellular dynamics and maintenance of CD8^+^ T_E/EM_ responses in spleen and liver depending on vaccination dose and frequency. While the spleen appeared to be critical as priming and source compartment of CD8^+^ T_E/EM_ responses, the formation of intrahepatic, protection-mediating T_RM_ cells was favored by booster injections and accelerated by higher dosing. Analysis by mathematical models further revealed an almost linear differentiation and migration pathway from splenic precursors toward hepatic memory cells during priming, providing a mechanistic framework for CD8^+^ T cell differentiation and proliferation by considering the distinct roles, but also the interplay of the spleen and the liver in the formation of protective immunity.

## Materials and Methods

### Mice and Parasites

Female C57BL/6 mice were obtained from Janvier Labs (Paris, France). NMRI mice were obtained either from Janvier or Charles River Labs (Sulzfeld, Germany). All mice were maintained under specific pathogen-free conditions. At the start of individual experiments mice were age-matched and 6–8 weeks of age.

All experiments were carried out with the rodent parasite *Plasmodium berghei* ANKA (*Pb*ANKA). *Pb*ANKA sporozoites were isolated by dissection of salivary glands (SGs) from female *Anopheles stephensi* mosquitoes at days 17–21 after a bloodmeal on infected NMRI mice. To obtain *Pb* ANKA radiation-attenuated sporozoites (*Pb*RAS), isolated sporozoites were treated by exposure to 150 Gy of X-rays (X-rad 320, Precision X-Ray, University Hospital Heidelberg).

### Immunization and Challenge Experiments

**Figure 1:**
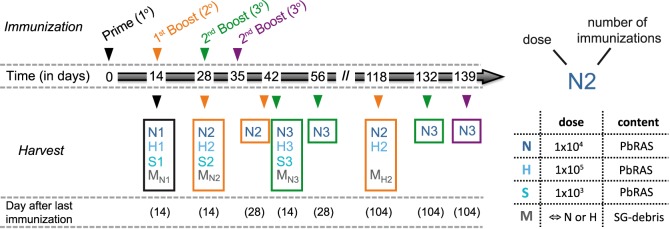
Immunization and analysis schedule. Schematic indicating the time points at which mice were vaccinated and analyzed according to various immunization protocols. Mice received either only a prime (1°, black), a prime-boost (2°, orange), or a prime-boost-boost vaccination scheme (3°, green) with 14 day-intervals between subsequent immunizations using three doses of *Plasmodium berghei* RAS (*Pb*RAS) including 1 × 10^3^ (S, sub-protective), 1 × 10^4^ (N, normal), and 1 × 10^5^ (H, high). For one additional group (3°, purple), the second booster injection was administered 21 days after the last boost. Mock-control (M) groups received corresponding doses of salivary gland (SG) debris. Mice were analyzed at indicated timepoints with the time in days after the last immunization noted. Each group is characterized by the dose and the number of injections received and comprised *n* = 3–6 animals. A detailed table comprising all individual group sizes is shown in Table S1 in Supplementary Material.

For all immunizations C57BL/6 mice received intravenous (i.v.) injections either with three different doses of *Pb*RAS or an equivalent mock dose of irradiated SG homogenate from uninfected mosquitoes that received a bloodmeal from naïve NMRI mice. All i.v. injections were delivered in a total volume of 100 µl PBS shortly after irradiation using either 1 × 10^3^ (S-dose), 1 × 10^4^ (N-dose), or 1 × 10^5^ (H-dose) *Pb*RAS. For immunization doses and timings refer to Figure 1 with individual group sizes specified within Table S1 in Supplementary Material.

Mice were challenged by i.v. injection of 1 × 10^3^ infectious *Pb*ANKA sporozoites in a total volume of 100 µl PBS and examined daily for parasitemia by Giemsa-stained smears from tail blood, starting at day 3 after challenge. In one set of experiments, mice that were protected from a previous challenge received a second i.v. challenge (re-challenge) with 1 × 10^4^ *Pb*ANKA sporozoites. Protection was defined as the absence of blood-stage parasites by days 14–28 post-challenge.

### Cell Preparation

At defined time points (see Figure 1) mice were euthanized by CO_2_ inhalation. Spleens and livers were removed following perfusion of the liver with 10 ml PBS. Total splenocyte or liver cell suspensions were obtained by passing the organs through a 70-µm nylon cell strainer (BD Biosciences) or a fine metal strainer (250 µm), respectively. Cells were washed with complete RPMI 1640 medium (RPMI supplemented with 10% FCS, 1× MEM NEAA, 1 mM sodium pyruvate, 5 ml penicillin/streptomycin; all from Gibco). The liver cell suspension was mixed with 36% Easycoll (Biochrom AG) and spun for 10 min, 520 × *g* at room temperature. For both preparations (liver and spleen), erythrocytes were lysed for 5 min on ice with lysis buffer (0.037 g EDTA, 1 g KHCO_3_, 8.26 g NH_4_Cl in 1 l ddH_2_O, pH 7.4). Subsequently cells were washed with complete medium and counted in Trypan blue.

### Cell Staining, Antigen-Specific Stimulation, and Flow Cytometry

Isolated cells from spleen and liver tissue were labeled with monoclonal antibodies (eBioscience): Fluorescein isothiocyanate-conjugated anti-CD8 (53-6.7), allophycocyanin (APC)-conjugated anti-CD44 (IM7), Peridinin Chlorophyll Protein-Cyanine5.5 (PerCP Cy5.5)-conjugated anti-CD62L (MEL-14), phycoerythrin-conjugated anti-IFN-γ (XMG 1.2), phycoerythrin-Cyanine7-conjugated anti-CD69 (H1.2F3). For all stainings, anti-CD16/CD32 (96) was added to block Fc receptors.

Briefly, surface staining was performed in PBS containing monoclonal antibodies for 20 min on ice. Intracellular staining (ICS) was only done following antigen-specific stimulation (see below). For ICS, cells were washed with PBS before fixation with 2% PFA/PBS for 15 min at room temperature followed by staining with anti-IFNγ antibody in permeabilization buffer (0.1% BSA, 0.3% Saponin in PBS) for 20 min on ice. Finally, cells were washed and re-suspended in PBS (subsequent data acquisition) or 1% PFA/PBS, incubated for 5 min at room temperature in the dark, washed once with PBS and stored at 4°C until data acquisition. Among the CD8^+^ T cells, we distinguished between T_N_ (naïve; CD44^lo^/CD62L^hi^), T_CM_ (central memory; CD44^hi^/CD62L^hi^), T_E/EM_ (effector/effector memory; CD44^hi^/CD62L^lo^), and T_RM_ (resident memory; CD44^hi^/CD62L^lo^/CD69^hi^) cells according to their surface markers (Figure S1 in Supplementary Material).

For the analysis of the antigen-specific response to the peptide SALLNVDNL (*Pb*T130) of the *Pb*TRAP protein (25) cells were incubated in complete medium for 16 h at 37°C in the presence of 1 µM *Pb*T130 and Brefeldin A (Sigma-Aldrich, 10 µg/ml) prior to cell staining as described. Cells were measured using a FACSCanto I flow cytometer (BD Bioscience).

### Data Analysis and Statistics

Flow cytometry data were analyzed using FlowJo software (version 10.1; FlowJo LLC). Total cell numbers per organ following direct *ex vivo* surface staining and FACS analysis were calculated by relating percent of the respective cell subset of total detected events to the cell numbers obtained after cell preparation and counting. To calculate total numbers of *Pb*T130-specific CD8^+^ T_E/EM_ cells per organ, the percentage of IFN-γ-positive (IFN-γ^+^) cells of CD8^+^ T_E/EM_ cells measured after 16 h *Pb*T130-stimulation and ICS was related to the total number of CD8^+^ T_E/EM_ cells obtained directly *ex vivo* following surface staining assuming equal loss rates for cells during overnight-stimulation. Statistical analysis was performed using nonparametric rank-based relative comparison adjusted for multiple comparisons based on the *mctp*-function in the R-language of statistical computing (26) to account for small group sizes. *p*-values with *p* < 0.05 were considered statistically significant.

### Mathematical Model to Describe Booster Effects on Cell Populations Within the Spleen

We developed a simple mathematical model to describe the effect of each prime and booster injection on the number of T_CM_ and T_E/EM_ cells in the spleen 14 days after the last injection. Assuming that the specific cell subpopulation in the spleen, *T*, increases after each injection, *b*, by a dose-dependent factor λ*_d_*, the concentration of cells after *b* + *1*-injections is then defined by

(1) $$T(b+1,d)=(1+\lambda_{d})T(b,d)\;\text{with}\;b=0,1,2,\ldots .$$

Hereby, *T*(0) = *T*_0_, defines the number of cells within the particular CD8^+^ T cell subpopulation before prime. While Eq. 1 assumes that the increase in the specific CD8^+^ T cell subpopulation in the spleen is independent of other compartments, we also extended the model by assuming that additional cell growth in the spleen decreases dependent on the level of T_E/EM_ cells in the liver, *T_L_*, thus

(2) $$T(b+1,d)=\left(1+\lambda_{d}\frac{T_{L}^{\tau }(b,d)}{T_{L}^{\tau }(b,d)+\Omega_{L}^{\tau }}\right)T(b,d)\;\text{with}\;b=0,1,2,\ldots .$$

Hereby, Ω*_L_*, defines the number of T_E/EM_-cells in the liver at which the additional increase is half of the maximum, and τ a Hill-coefficient indicating the steepness of the assumed saturation effect (see also Appendix A1 in Supplementary Material).

We fitted Eqs. 1 and 2 to the experimental data of T_CM_ and T_E/EM_ cells in the spleen estimating the initial number, *T*_0_, the dose-dependent increase per boost, λ*_d_*, and λ*_d_* and Ω*_L_*, respectively. For Eq. 2, we used the mean values for the measured number of T_E/EM_ cells in the liver and tested different assumptions for the Hill-coefficient τ. Comparison of model performance was assessed by the corrected Akaike information criterion (AICc) (27).

### Mathematical Model Describing CD8^+^ T Cell Differentiation Dynamics in Spleen and Liver

We developed a mathematical model to describe the individual cellular subset dynamics and their interactions in response to different vaccination schemes. The mathematical model is explained in detail in Appendix A2.1 in Supplementary Material. In brief, the modeling framework based on ordinary differential equations considers the proliferation and migration dynamics of five different cellular subtypes including naïve, T_CM_ and T_E/EM_ cells in the spleen, as well as T_E/EM_ and T_RM_ cells in the liver. T_CM_ cells in the liver were neglected as we did not see any influence of immunization protocols on these numbers, suggesting that their appearance in the liver is mainly by chance and dependent on their concentration in the spleen. The modeling framework allows the consideration of all possible interactions and differentiation pathways between the different cellular compartments. We used an unbiased model selection algorithm (Appendix A2.2 in Supplementary Material) to determine the relevant processes and differentiation pathways by fitting the model to the experimental data of all N-dose vaccinated groups based on a maximum likelihood approach. To analyze the dynamics of the vaccination-induced responses, data were normalized by the corresponding measurements for each cellular subset within naïve mice. Model performance was assessed using the corrected AIC (AICc). Identifiability of parameter estimates was assessed using profile likelihood analysis (28) with estimates shown in Figure A2.2 and Table A3.1 in Supplementary Material. All analyses were performed using the R language of statistical computing (26).

## Results

### CD8^+^ T Cell Numbers in the Liver Increase Substantially After Booster Injections During Variable *Pb*RAS Vaccination Regimens

In order to examine the effect of variable vaccination regimens on the development of CD8^+^ T cell responses, we immunized groups of C57BL/6 mice by intravenous (i.v.) injection of *Pb*RAS varying the vaccine dose, number of injections, and time to analysis (Figure 1). A total of three different doses were analyzed, including a “normal” (N-dose: 1 × 10^4^), a “sub-protective” (S-dose: 1 × 10^3^), and a “high dose” (H-dose: 1 × 10^5^) of *Pb*RAS.

**Figure 2:**
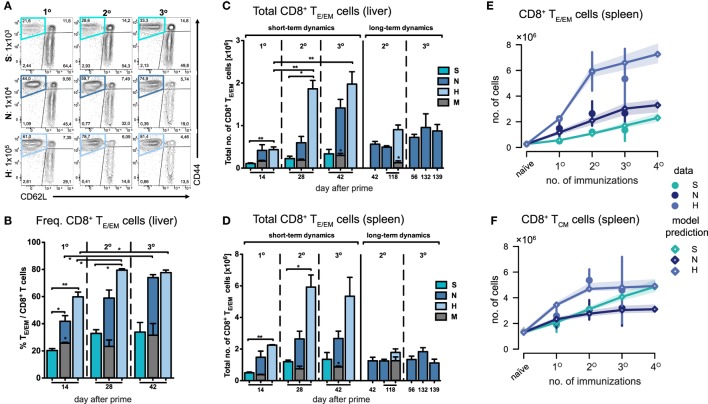
Dynamics of CD8^+^ T_E/EM_ cells after varying *Plasmodium berghei* RAS vaccination protocols. **(A)** Representative FACS-plots of CD8^+^ T cell responses gated for CD62L and CD44 measured in the liver of mice receiving prime (1°), prime-boost (2°), or prime-boost-boost (3°) immunizations with S-, N-, or H-dose. **(B)** Increasing percentage of T_E/EM_ cells among CD8^+^ T cells in the liver with subsequent booster injections dependent on the vaccination dose. Corresponding total number of T_E/EM_ cells in the liver **(C)** and spleen **(D)** looking at short-term (measurements taken 14 days after last injection) and long-term dynamics (>14 days after last injection). Numbers below the plots indicate time of measurement in days post prime. Numbers of animals per group are specified within Table S1 in Supplementary Material. Graph bars depict means with SEM; *p < 0.05; **p < 0.01; ***p < 0.001; multiple nonparametric rank-based relative comparison. **(E,F)** Measured and predicted levels of T_E/EM_ **(E)** and T_CM_ cells **(F)** in the spleen using a mathematical model that assumes splenic reactivation to depend on the level of hepatic T_E/EM_ cells prior to immunization. Shaded areas indicate the 95%-confidence intervals of model predictions.

Measuring cell numbers 14 days after the last immunization, we found that both the frequency and total number of T_E/EM_ (CD44^hi^/CD62L^lo^) cells in the liver increased with respect to the vaccine dose and the number of injections given (Figures 2A–C). Interestingly, the magnitude of the primary response in the liver measured in absolute cell numbers 14 days after prime did not significantly differ between the N- and H-dose (Figure 2C). However, a notable difference was observed after the first boost, which led to an about threefold higher total number and increased frequency of T_E/EM_ cells in H-dose immunized animals (N2: 0.6 ± 0.15 × 10^6^ and H2: 1.86 ± 0.2 × 10^6^ total T_E/EM_ cells). For the standard immunization with the N-dose we observed that only the second boost (N3) led to a substantial increase in the total T_E/EM_ cell numbers reaching a level of 1.42 ± 0.2 × 10^6^ cells compared to 0.42 ± 0.13 × 10^6^ (N1) and 0.6 ± 0.15 × 10^6^ (N2) cells after prime and first boost, respectively. Here, cell levels obtained after three immunizations with the normal dose are comparable to those that are already reached after one boost with the H-dose (H2) (Figures 2B,C). Thus, a total amount of 3 × 10^4^ *Pb*RAS divided into three i.v. injections (N3) produced similar quantities of T_E/EM_ cells in the liver as detected in mice that received an almost sevenfold higher absolute number of *Pb*RAS administered through only two i.v. injections (H2). While booster effects were much less pronounced following S-dose immunizations, which generated significantly lower proportions of T_E/EM_ cells compared to immunizations by N- and H-doses (Figures 2B,C), the H-dose prime-boost regimen (H2) apparently resulted in a saturation of the T_E/EM_ level in the liver (79.5 ± 1.02% of total CD8^+^ T cells) that was not further elevated by additional boosting (H3), neither in frequency nor absolute numbers. Analysis of the hepatic CD8^+^ T cell subsets further revealed that only a small fraction of around 10% of cells exhibited a T_CM_ phenotype (CD44^hi^/CD62L^hi^), which was seemingly unaffected from the choice of dose and number of injections (Figure S2 in Supplementary Material).

In summary, we observed short-term cumulative booster effects for immunizations with N- and H-doses, but we found no linear relationship between the dose or the number of injections received and the resulting hepatic T_E/EM_ levels.

### CD8^+^ T Cell Dynamics in the Spleen Are Largely Unaffected After Two Immunizations and Influenced by T_E/EM_ Cell Levels Generated in the Liver

In comparison to the liver, T_E/EM_ frequencies in the spleen were much lower, even though the total number of cells was substantially higher (Figure 2D; Figure S3 in Supplementary Material). Most prominently, H-dose immunization led to levels of around three times the amount of the respective hepatic subpopulation 14 days after the first or second boost (H2: 5.92 ± 0.77 × 10^6^ and H3: 5.34 ± 1.2 total T_E/EM_ cells). For all different doses, in particular for the H-dose, the strongest increase in T_E/EM_ cell numbers occurred after the first boost, after which both the T_E/EM_ frequencies and the total number of cells stabilized at levels which were not affected by additional booster injections. This was even true for the normal dose, for which a substantial increase in hepatic T_E/EM_ numbers was observed in response to a second boost (Figures 2C,D). Similar observations were made for the central memory subset, with the number of CD8^+^ T_CM_ cells in the spleen following similar dynamics than the splenic T_E/EM_ subset, in particular for the N- and H-doses.

Analyzing possible interactions between spleen and liver during immunizations, we found that a mathematical model with the assumption that the dose-dependent increase of T_E/EM_ cells in the spleen is negatively impacted by higher prior-levels of T_E/EM_ cells in the liver at the time of boosting (see Materials and Methods) fitted the data significantly better than a model assuming no interaction between both compartments [AICc: 26.8 (dependent) vs. 39.5 (independent), Figure 2E]. We estimated that N- and H-dose would lead to a maximal additional 3.4- (2.5, 4.6) and 7.4-fold (6.1, 9.2) increase, respectively, in the number of T_E/EM_ cells in the spleen 14 days after injection, compared to an only 1.1-fold (0.8, 1.4) additional increase using the S-dose (see Appendix A1, Table A1.1 in Supplementary Material, numbers in brackets define 95%-confidence intervals of estimates). In addition, we found that an estimated level of around 2.3 × 10^5^ T_E/EM_ cells in the liver reduces the activation of CD8^+^ T cells in the spleen by 50%. As expected from the data, we only estimate a moderate additive effect of booster injections on the number of T_CM_ cells in the spleen (Table A1.1 in Supplementary Material; Figure 2F).

Thus, as for the liver, we also observed dose-dependent effects on the splenic T_E/EM_ levels with subsequent immunizations but with no further increase in cellular numbers after more than one booster injection, which was irrespective of the given dose and could be explained by the previously generated T_E/EM_ levels in the liver.

### Dynamics of Antigen-Specific CD8^+^ T Cell Responses in Spleen and Liver Indicate Increasing Antigen-Specificity With Subsequent Booster Injections

**Figure 3:**
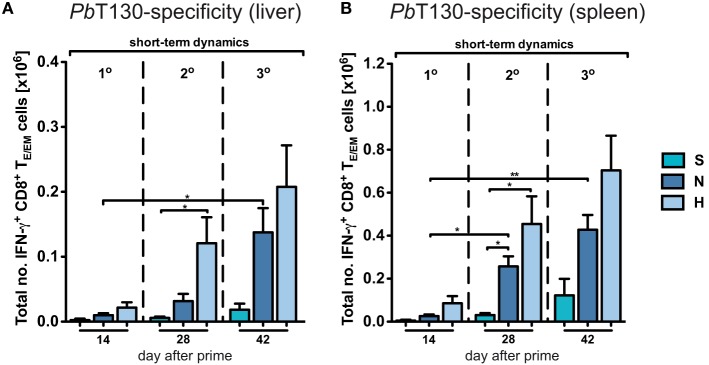
Dynamics of antigen-specific CD8^+^ T_E/EM_ cells after varying *Plasmodium berghei* RAS vaccination protocols. Antigen-specificity was measured by IFN-γ expression of CD8^+^ T_E/EM_ cells following overnight-stimulation with *Pb*T130. The number of IFN-γ expressing T_E/EM_ cells in the liver **(A)** and spleen **(B)** of mice receiving different vaccination protocols was determined based on the measured frequencies after overnight-stimulation (Figure S3 in Supplementary Material). Numbers below the plots indicate the time point of measurement in days post prime. Numbers of animals per group are specified within Table S1 in Supplementary Material. Graph bars depict means with SEM; *p < 0.05; **p < 0.01; ***p < 0.001; multiple nonparametric rank-based relative comparison.

Due to the general paucity of malaria-specific CD8^+^ T cell epitopes identified (25, 29), we concentrated our analyses on the dynamics of the total CD8^+^ T cell response in the context of different immunization schemes. Comparing them to the responses in a corresponding mock-control group (M_N_), we found that hepatic T_E/EM_ cells remained relatively stable at low levels throughout the vaccination schedule and constituted only about 20% of the T_E/EM_ numbers detected in the respective *Pb*RAS immunized mice after three i.v. administrations (Figure 2C). Notably, total T_CM_ cell numbers did not significantly differ between the mock-control group and mice vaccinated with any other dose, therefore preventing conclusions about *Pb*RAS-specific T_CM_ responses in liver and spleen (Figures S2A–D in Supplementary Material). In order to also assess the dynamics of antigen-specific CD8^+^ T cells, we re-stimulated isolated lymphocytes with the antigenic peptide SALLNVDNL (*Pb*T130) of the *Pb*TRAP protein and measured intracellular IFN-γ that was specifically secreted by T_E/EM_ cells of *Pb*RAS immunized mice (25). Following three i.v. injections with the N- or H-dose, about 10 or 15% of the overall T_E/EM_ response in liver or spleen, respectively, appeared to be *Pb*T130-specific (Figure S4 in Supplementary Material). Extrapolating these frequencies to the total number of antigen-specific T_E/EM_ cells, the dynamics of the antigen-specific T_E/EM_ response showed a similar pattern as the overall short-term response, however, with a more linear increase in antigen-specific T_E/EM_ cell numbers with respect to the dose and number of injections (Figure 3). In contrast to the overall response our data do not indicate a saturation of *Pb*T130-specific T_E/EM_ cells after multiple immunizations in spleen or liver. This suggests that the composition of the total response is still changing while the magnitude remains constant.

### The Level of Short-Term Protection Differs Between Different Vaccination Schemes

**Figure 4:**
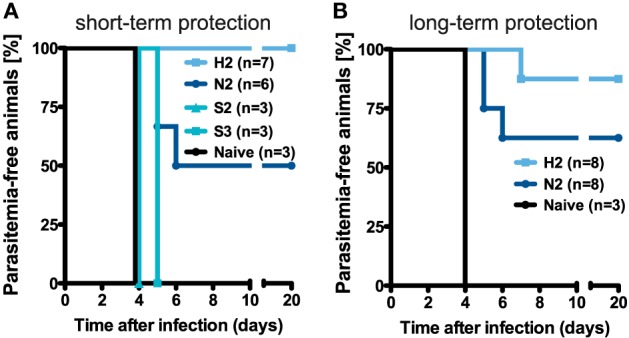
Protective efficacy of different vaccination regimens. **(A)** Schematic of the protocol determining the groups of mice that were challenged. **(B,C)** Percentage of protected animals receiving either two or three injections with the N-, H-, or S-dose, respectively. Mice were challenged by i.v. injection of 1 × 10^3^ infectious *Pb*ANKA sporozoites in a total volume of 100 µl PBS on day 14 [**(B)**, short-term] and more than 100 days post last injection [**(C)**, long-term] with protection defined as the absence of blood-stage parasites. See also Table S2 in Supplementary Material.

To compare the efficacy of the previously applied immunization protocols in mediating protection, groups of mice were challenged with 1 × 10^3^ infectious sporozoites after receiving different numbers of immunizations. Based on previous analyses showing that three i.v. injections with the normal dose of 1 × 10^4^ *Pb*RAS confer long-lasting sterile protection against challenge with infectious sporozoites (19, 30, 31), we were interested to compare the level of protection that is potentially mediated by the measured secondary T_E/EM_ responses following prime-boost immunizations. While secondary responses in H-dose injected mice protected all animals challenged 14 days after the last immunization (*n* = 7 of 7), two *N*-dose injections induced a protective level of only 50% (*n* = 3 of 6) with a mean prepatent period of 5.3 days (Table S2 in Supplementary Material). In contrast, none of the S-dose immunization regimens resulted in protection (Figure 4A). Thus, the level of protection seems to depend on the level of T_E/EM_ cells in the liver.

### Long-Term Dynamics Show Decreasing CD8^+^ T Cell Levels in Liver and Spleen While Protection Levels Are Maintained

We next addressed the question how secondary and tertiary responses progress over time and if secondary responses induced by N- and H-dose immunizations are stable and able to confer protracted protection. Following N-dose prime-boost immunization (N2) for more than 100 days after prime, we found that the long-term T_E/EM_ pool in the liver remained mostly constant (Figure 2C; Figure S3A in Supplementary Material). In contrast to this, numbers of hepatic T_E/EM_ cells in the H2-dose group decreased considerably compared to the short-term response (H2_short-term: 1.86 ± 0.2 × 10^6^ and H2_long-term: 0.9 ± 0.11 × 10^6^ cells) but remained higher compared to the numbers detected after N2-immunizations (N2_long-term: 0.49 ± 0.031 × 10^6^ cells). For mice receiving three immunizations with the normal dose (N3), the long-term T_E/EM_ pool also declined before stabilizing at about the same level as hepatic T_E/EM_ cells in H2-immunized mice, but still slightly higher than T_E/EM_ cell levels after N2-immunizations (Figure 2C).

In comparison to the liver, the observed long-term dynamics was different for the spleen where secondary and tertiary T_E/EM_ pools in N- and H-dose vaccinated mice all decreased to levels that were comparable to the generated number of T_E/EM_ cells after primary responses. In general, there was no difference in these numbers with respect to the dose, the number of boosts or even to M-dose immunizations (Figure 2D; Figure S3B in Supplementary Material), indicating that distinct processes are responsible for the homeostasis of T_EM_ cells in liver and spleen.

Challenging the mice with 1 × 10^3^ infectious sporozoites more than 100 days after the last immunization demonstrated that the protective efficacy of secondary responses was maintained over time (Figure 4B). We found that for N2-immunization five out of eight mice (62.5%) were protected, while an H2-immunization strategy resulted in sterile protection in seven out of eight mice (87.7%) with the one mouse that became blood-stage positive showing a delayed prepatent period of 7 days. Furthermore, all of the challenged blood-stage negative animals from the N- and H-dose groups also showed complete protection against an intravenous re-challenge with 1 × 10^4^ infectious sporozoites three weeks after the first challenge (Table S2 in Supplementary Material).

In agreement with previous studies that pointed out the importance of the hepatic CD8^+^ T_E/EM_ subset for protracted protection (16, 21, 24), our analysis of late secondary responses indicated a difference in hepatic but not splenic T_E/EM_ levels between mice that were highly protected (H2) and those displaying only intermediate protection (N2).

### Tissue-Resident Memory Cells Dominate the CD8^+^ T Cell Response in the Liver

**Figure 5:**
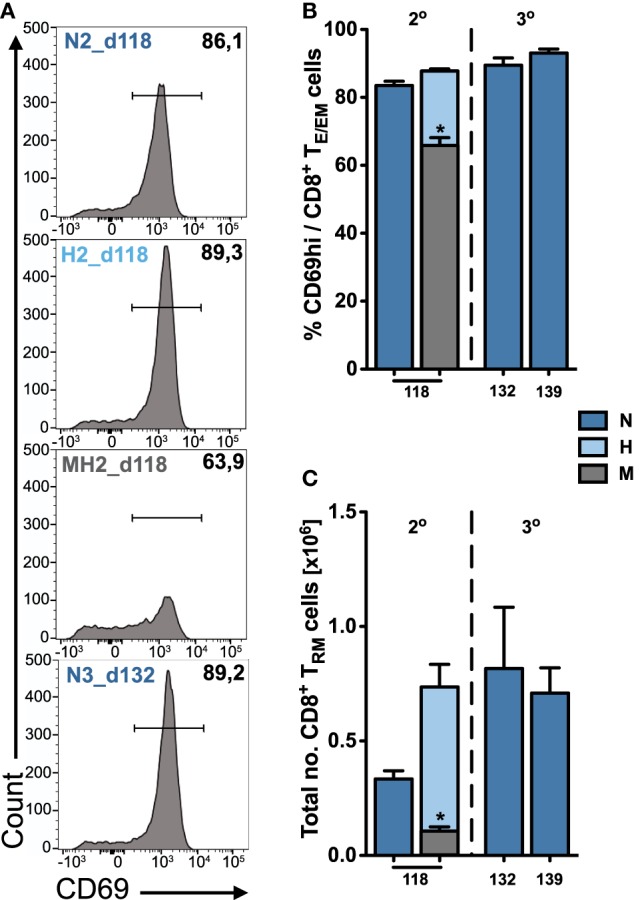
Quantification of tissue-resident CD8^+^ T_RM_ cells in the liver. **(A)** Representative FACS histograms showing the expression of CD69 on hepatic T_E/EM_ cells for long-term responses of vaccinated animals. Percentage **(B)** and total number **(C)** of tissue-resident CD8^+^ T_RM_ cells among T_E/EM_ cells indicating increasing numbers according to the dose and number of injections. Numbers below the plots indicate time of measurement in days post prime. Numbers of animals per group are specified within Table S1 in Supplementary Material. Graph bars depict means with SEM; *p < 0.05; **p < 0.01; ***p < 0.001; multiple nonparametric rank-based relative comparison.

As our data indicate an improved maintenance of hepatic compared to splenic T_E/EM_ cells, we looked more closely at the local CD8^+^ T_E/EM_ cell subsets in the liver. Therefore, we analyzed CD8^+^ T_E/EM_ cells for the expression of CD69, a T_RM_ marker used for various tissues, including most recently also for liver-specific localization of parasite-specific CD8^+^ T cells in *Pb*RAS immunized C57BL/6 mice (21, 24) (Figure 5A).

We found that long-term hepatic T_RM_ frequencies were only slightly increased following two or three H- and N-dose immunizations (H2, N3) compared to only two N-dose (N2) vaccinations (Figure 5B). However, a substantial difference was observed for the total numbers of T_RM_ cells that were about twofold higher in H2- or N3-immunized animals (H2: 0.74 ± 0.1 × 10^6^ and N3_d132: 0.82 ± 0.27 × 10^6^ cells) than to N2-immunized animals (N2: 0.33 ± 0.04 × 10^6^ cells) more than 100 days after prime (Figure 5C). In contrast to the liver, we did not detect any significant expression or upregulation of CD69 on CD8^+^ T_E/EM_ cells in the spleen, which is consistent with previous publications (20, 21).

Altogether, our results show that the composition of the hepatic long-term CD8^+^ T_E/EM_ pool is governed by cells exhibiting a T_RM_ phenotype. This stresses the importance of a liver-resident CD8^+^ T cell population needed for conferring long-lasting protection against malaria infection.

### Mathematical Modeling Reveals the Cell Differentiation Pathway and the Dominant Contribution of Local Reactivation During Booster Injections for the Formation of Hepatic T_RM_-Levels

**Figure 6:**
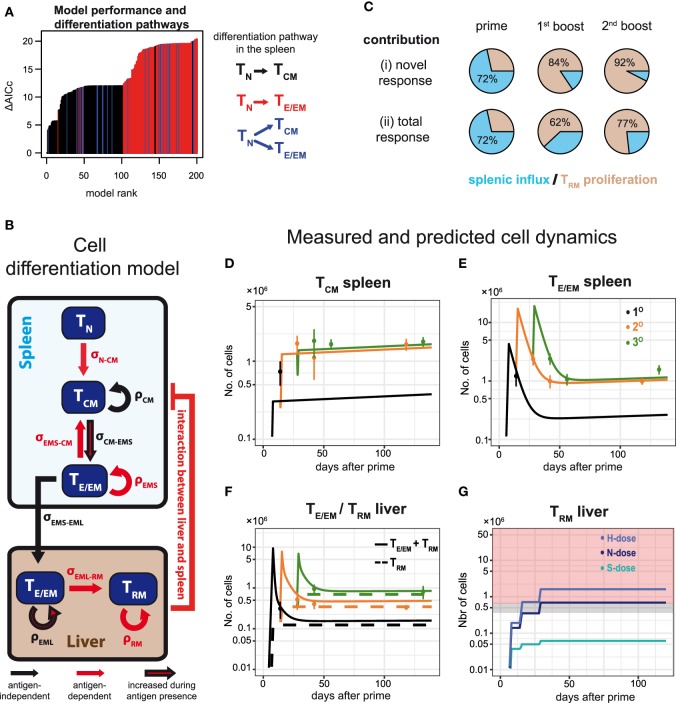
Examining CD8^+^ T cell differentiation dynamics in response to varying vaccination protocols using mathematical models. **(A)** The 200 best fitting models explaining CD8^+^ T cell activation and differentiation dynamics in N-dose vaccinated mice ranked according to the corrected Akaike Information criterion (AICc). Nearly all of the best performing models assume a linear T_N_–T_CM_ cell differentiation pathway (black) in the spleen. For a detailed explanation of the mathematical models and the unbiased model selection algorithm see Appendices A1 and A2 in Supplementary Material. **(B)** Schematic of the mathematical model best explaining the observed dynamics indicating proliferation, ρ, and cell migration/differentiation, σ, rates. Processes only active during the immunization periods are indicated in red. **(C)** Proportion of T_RM_ cells in the liver originating from local reactivation (ocher) or additional influx from the spleen (blue) after each prime and boost distinguished between the newly generated (i) and the total response (ii). **(D–F)** CD8^+^ T cell dynamics predicted by the model shown in **(B)** for the numbers of T_CM_ and T_E/EM_ cells in the spleen **(D,E)**, as well as the numbers for T_RM_ and the total T_E/EM_ cells (T_RM_ + T_EML_) in the liver **(F)** of N-dose vaccinated animals. Colored lines indicate the different vaccination protocols corresponding to prime (1°, black), prime-boost (2°, orange) or prime-boost-boost (3°, green). The corresponding measurements for the individual groups are shown as well (mean ± SEM). **(G)** Predicted dynamics of liver-resident T_RM_ cells for different doses according to the basic vaccination protocol. Gray and red shaded areas correspond to the number of T_RM_ cells required to mediate 50 and 100% protection, respectively, as determined by the challenge experiments.

In order to obtain a systematic and quantitative understanding on the influence of dosage and frequency of booster injections on the generation of potentially protective hepatic T_E/EM_ cells, we developed a mathematical model that describes the dynamics of the various CD8^+^ T cell subsets in more detail. Our mathematical model took into account all possible differentiation, migration and proliferation dynamics of the respective cell populations, as well as their interactions between spleen and liver (see Materials and Methods and Appendix A2.1 in Supplementary Material). We then used an unbiased model selection algorithm to identify the relevant processes that would best describe the experimental data (see Appendix A2.2 in Supplementary Material). Testing approximately 2,000 different models and ranking them by their ability to describe the experimental data, we identified one model that clearly outperformed the others when analyzing all N-dose vaccinated animals simultaneously (Figure 6A). This model assumes an almost linear differentiation pathway (Figure 6B): Upon priming, naïve cells first turn into T_CM_ cells in the spleen that differentiate further into T_E/EM_ cells, which migrate into the liver where they turn into T_RM_ cells. In general, nearly all highly ranked models support a naïve to T_CM_ differentiation pathway (T_N_-T_CM_) rather than a naïve to T_E/EM_ or a split differentiation pathway (Figure 6A). While proliferation of T_CM_ and differentiation from T_CM_ into T_E/EM_ cells in the spleen, as well as migration of T_E/EM_ cells to the liver and their subsequent proliferation is assumed to occur independent of antigen presence after the first antigen exposure, differentiation into T_RM_ cells in the liver is identified to be dependent on antigenic stimuli that are provided during priming and booster injections (Figure 6B). We also estimate that about 72 ± 3% of hepatic T_RM_ cells after priming originate from splenic precursors, while 84 ± 8% and 92 ± 4% of the additional T_RM_ cells after first and second booster injections, respectively, are generated by local reactivation (Figure 6C; Table S3 in Supplementary Material). Most of the model parameters describing cell net-proliferation and migration can be reliably quantified (Figure A2.2 in Supplementary Material), and a representative plot for the individual subset dynamics is shown in Figures 6D–F. Despite large differences in the prediction for T_E/EM_ cell numbers in spleen and liver during the expansion phases after priming and subsequent booster injections, for which no experimental data were available (Appendix A2.3 in Supplementary Material), all predictions for the long-term dynamics, including the generation of the T_RM_ pools in the liver, are robust independent of the selected parameter combination.

In addition, following the cellular dynamics identified for the N-dose vaccinated animals (Figure 6B), we studied the impact of varying vaccination doses on these dynamics by incorporating a dose-factor into our mathematical model (see Materials and Methods). We found that the cellular dynamics observed for both the high and the sub-protective dose could be explained mostly by a changed differentiation rate from T_CM_ to T_E/EM_ in the spleen and a dose-dependent change in the T_RM_ net-proliferation rate (Appendix A3 in Supplementary Material). A high dose leads to an estimated 1.2-fold increase in these two rates compared to the normal dose, while for the sub-protective dose we estimate a reduction to around 20% of their baseline values, which explains the failure of this dose to mount a robust T_RM_ response in the liver (Figure 6G).

Thus, our mathematical analysis supports the reversing roles of splenic and hepatic responses during subsequent immunizations, with re-expansion of local T_RM_ cells dominating the generation of protective T_RM_-levels during booster injections, therefore, emphasizing the need of high-dose booster injections.

## Discussion

Understanding the dynamics that shape the development of protective immune responses during immunization is an important prerequisite to design effective vaccination strategies. For malaria, the implementation of promising whole sporozoite vaccination approaches into clinical practice still lacks the identification of appropriate protocols as the correlates of protection and the effect of dosage and frequency of injections on their generation need to be determined (13). In animal models, it has been shown that CD8^+^ T cells play a critical role in mediating immunity making them an important target for vaccine development (9, 21, 22). Here, using an extensive experimental protocol, we systematically analyzed the impact of various prime-boost vaccination regimes on the dynamics of CD8^+^ T cell responses in spleen and liver and their influence on protection.

Our data corroborate a critical role for the spleen during priming for effective RAS immunization *via* the intravenous route. Previous studies already showed that the formation of protective immunity against malaria infection was hampered in splenectomized mice (32), and that the spleen represents the main priming site of vaccine-induced responses by splenic CD8α^+^ dendritic cells (21, 33). In line with these findings, we observed that splenic CD8^+^ T cell responses mainly develop during the first two immunizations and are less affected by subsequent booster injections. Our mathematical analysis indicated that this reduced accumulation of T_E/EM_ cells in the spleen by booster immunizations can be explained by the hepatic T_E/EM_ levels obtained during previous vaccinations (Figure 2E). Probably the increased accumulation of tissue-associated CD8^+^ T cells at the site of infection in the liver makes further involvement of the spleen for systemic immune activation obsolete.

The involvement of the liver or its associated draining lymph nodes in the priming of CD8^+^ T cells after the first immunization seems to be minor but cannot be totally excluded (33). However, our observations suggest an increasing involvement of the liver for the generation of immunity with subsequent booster immunizations, mainly due to antigen-specific reactivation of preformed hepatic T_EM_ and T_RM_ pools. Based on a mathematical model that describes cell differentiation and proliferation in response to immunizations, we estimate that on average ~84–94% of all newly formed T_RM_ cells in the liver originate from proliferation of locally re-activated CD8^+^ T cells during booster injections. Migration of splenic T_E/EM_ cells towards the liver, which dominated the T_RM_-population during priming (~70–75% of cells) seems to only play a minor role at this point (Table S3 in Supplementary Material). Thus, the importance of spleen and liver for mounting protective responses reverts with subsequent booster injections, which is in line with recent findings that show the dominant impact of tissue-resident memory T cells for the generation of secondary memory responses (34, 35).

The increasing involvement of the hepatic CD8^+^ T cell response might be supported by continuously accumulating, activated dendritic cells at the site of infection, as shown to occur in the course of i.v. RAS immunizations and other infection models (23, 36–40). Enhanced frequencies of antigen-presenting cells (APC) in the liver might promote the reactivation and build-up of local hepatic CD8^+^ T cell populations during vaccine-induced secondary and tertiary responses.

Importantly, our results show that the accumulation of hepatic CD8^+^ T cells during booster immunizations appeared to be dose-dependent. While for the H-dose, T_E/EM_ cells in the liver already reached a plateau after the first boost, T_E/EM_ levels still increased for N-dose immunized animals with a second booster injection. In line with these observations, our mechanistic model also predicted dose-dependent effects to mainly affect the generation of hepatic CD8^+^ T cells, i.e., estimating an increase in both T_RM_ proliferation rates and the conversion of intrahepatic T_E/EM_ to T_RM_ cells for H-dose compared to N-dose immunizations (Appendix A3 in Supplementary Material). A higher immunization dose might cause a stronger inflammatory reaction in the liver thereby accelerating local immune activation and promoting the reversal of the prevailing state of immune-tolerance (18, 41). Notably, a recent study reported that the accumulation and activation status of intrahepatic CD8α^+^ DC was significantly increased by i.v. injection of a higher compared to a lower *Pb*RAS dose (40).

A change in the hepatic immune milieu accompanied by more frequent antigen-encounter/recognition in the liver potentially also drives the formation of tissue-resident CD8^+^ T_RM_ cells (21, 42, 43). Indeed, using CD69 as a marker for tissue-residency, we observed an increased accumulation of CD69^+^ T_E/EM_ (T_RM_) cells in the liver with subsequent booster injections. Although these hepatic T_RM_ cells might have some potential to reenter the circulation as recently demonstrated for thymic CD69^+^CD103^−^ memory CD8^+^ T cells (44), the observation of an increased accumulation of this cell subset in the liver emphasized the general appropriateness of this marker (21, 43, 45).

The generation of sufficient tissue-resident CD8^+^ T cells in the liver seems to be a key for developing protective immunity against malaria, increasing the need for understanding their differentiation dynamics (21, 46). However, the differentiation of T cells in response to immunization and infection is still highly debated. While some studies point toward a differentiation pathway following naïve-T_CM_-T_E/EM_ for CD8^+^ (47) and CD4^+^ T cells (48), other studies suggest inverse differentiation pathways or T_CM_ and T_E/EM_ cells to be two separate lineages of development (49–51). Performing a systematic analysis of all possible cellular interactions, our analysis supports a differentiation pathway following naïve-T_CM_-T_E/EM_ and subsequent T_RM_ generation (Figure 6B) corresponding to the findings by Buchholz et al. (47). Differences to other differentiation pathways identified previously could be due to the varying sites at which cells were sampled. Furthermore, our analysis might also be influenced by the requirement to simultaneously analyze priming and booster differentiation pathways, as during booster immunizations a T_CM_-T_E/EM_ differentiation is likely to be expected (52). Thus, a more detailed analysis of the cellular expansion phase after priming is needed to improve the characterization of the differentiation pathway, and to further determine the processes and factors leading to T_RM_ generation (43, 53).

Despite the complexity of our model in the context of the available data, estimates for all antigen-independent parameters, which determine the long-term dynamics of the response, are remarkably robust (see Appendix A2 in Supplementary Material). Most of the parameters that could not be identified are related to the expansion phase of the response directly after priming and booster injections. Identification of these antigen-dependent parameters is especially important to predict the influence of variations in the timing of immunizations on the generation of protective T cell responses. Using mathematical modeling, a previous study on the interaction of WSV dose and the timing of booster injections recently reported that boosting during the late phase of clonal contraction maximized memory T cell formation when using lower RAS doses, while a single inoculation was more effective in this respect when using a higher dose (54). However, this study only considered administration of a single boost not later than 7 days post priming and did not take into account the impact on interacting organ-specific or late memory responses. Obtaining more detailed information on organ-specific CD8^+^ T cell subset dynamics during the expansion phase will be essential to improve the characterization of the differentiation dynamics and consequently also to optimize the timings of immunizations.

Furthermore, the general lack of pre-erythrocytic, particularly protection-associated, epitopes hinders a comprehensive analysis of malaria-specific responses. Therefore, our study mainly concentrated on the dynamics of the vaccination-induced total responses. Although the measured responses likely contain activated CD8^+^ T cells induced by non-malaria antigens or bystander effects, our data indicated a clear vaccination-induced dynamics, as, e.g., shown by the lower cell levels in the corresponding Mock-control groups (Figures 2C,D). In addition, the increase in cellular numbers by booster injections corresponded to an increase in the frequency of antigen-specific CD8^+^ T cells according to TRAP130 re-stimulation (Figure 3). Remarkably, this increase in T130-specific CD8^+^ T cell numbers in spleen and liver by consecutive boosting also occurred despite the total number of CD8^+^ T cells becoming saturated. Thus, although there is indication for a maximal “carrying capacity” of T_E/EM_ and T_RM_ cells, the composition of the response is still changeable as observed in prime-boost vaccination regimes for other pathogens (55, 56). In fact, it was demonstrated that only T130-specific responses were recalled by homologous *Pb*RAS boosting in C57BL/6 mice, while responses to other antigens contracted, similar to results obtained for CSP in the BALB/c mouse model (57, 58). To which extent factors such as antigen abundance, localization and time of expression collectively influence specific pre-erythrocytic CD8^+^ T cell responses and shape the composition of WSV-induced recall responses remains to be determined (38, 57, 59–61). In this context, it also remains an interesting and open question to which extent sporozoite, liver stage or stage-transcending T_RM_ and T_E/EM_ responses contribute to protective immunity.

In summary, our analysis suggests a reversing role of spleen and liver during priming and subsequent booster injections following i.v. *Pb*RAS immunization. Booster injections are supposed to predominantly reactivate intrahepatic CD8^+^ T cell pools and are essential for the generation of a protective, mainly self-sustaining T_RM_-population, with higher dosing accelerating this process. Prime-and-trap vaccination strategies as applied before can exploit this mechanism efficiently (21). To which extent different routes of immunization (e.g., subcutaneous vs. intravenous) (39), as well as supportive drug treatment (62) might additionally influence the dose-dependent generation of protective T_RM_ cells remains to be investigated. A recent study already provided evidence that decreased protection following administration of sporozoites into the skin compared to i.v. injection is not linked to a lower parasite liver load, but rather favors the induction of regulatory immune responses in the liver and skin-draining lymph nodes with a negative impact on memory CD8^+^ T cell responses (39). Addressing this aspect within a mechanistic and quantitative analysis as presented here, as well as identifying the role of hepatic CD8^+^ T cells for protection against malaria infections in humans (13), can help to translate these findings made in murine models to clinical application for evaluating and informing the development of effective vaccination strategies (63). Our analysis provides a first mechanistic framework to describe the induction of protective hepatic CD8^+^ T cell responses against malaria. Identification of novel malaria-specific epitopes, as well as additional information on the expansion dynamics, will help to improve this model to explain dosage dependent vaccination effects on antigen-specific responses. As such it provides an important prerequisite for the rational design of effective immunization protocols and vaccination strategies against *Plasmodium*-induced infections.

## Ethics Statement

All animal experiments were performed according to FELASA category B and GV-SOLAS standard guidelines. Animal experiments were approved by the German authorities (Regierungspräsidium Karlsruhe, Germany), § 8 Abs. 1 Tierschutzgesetz (TierSchG) under the license G-258/12. No human samples were used in this study. All experiments were carried out according to standard institutional biological and gene technology safety guidelines.

## Author Contributions

Conceived the study: A-KM and FG; designed the study: RF, MG, A-KM, and FG; performed the experiments: RF and KH; developed the mathematical models and analysis methods: MG and FG; Analyzed the experimental data: RF, MG, A-KM, and FG; Wrote the manuscript: RF, MG, A-KM, and FG.

## Conflict of Interest Statement

The authors declare that the research was conducted in the absence of any commercial or financial relationships that could be construed as a potential conflict of interest.
