# Supplementary material for: Varying Immunizations With Plasmodium Radiation-Attenuated Sporozoites Alter Tissue-Specific CD8+ T Cell Dynamics
Source: Front Immunol. 2018 May 28;9:1137. doi: 10.3389/fimmu.2018.01137 (PMC5985394; doi:10.3389/fimmu.2018.01137)
Supplement: Supplementary file 1 [file presentation_1.PDF]

Varying immunizations with Plasmodium radiation-attenuated sporozoites alter tissue-specific CD8^{+} T cell dynamics

—

Supplemental Material

Roland Frank[1], Michael Gabel[2], Kirsten Heiss[1], Ann-Kristin Mueller[1,3,$,*], and Frederik Graw[2,$,*]

[1]Centre for Infectious Diseases, Parasitology Unit, University Hospital Heidelberg, Heidelberg, Germany
[2]Centre for Modeling and Simulation in the Biosciences, BioQuant-Center, Heidelberg University, 69120 Heidelberg, Germany
[3]German Center for Infection Research (DZIF), Heidelberg, Germany
[$]These authors contributed equally
[*]corresponding author: frederik.graw@bioquant.uni-heidelberg.de; ann-kristin.mueller@uni-heidelberg.de

Outline

Table S1 Experimental Groups

Table S2 Protection of mice against challenge with *Pb*ANKA sporozoites

Figure S1 Gating strategy used to analyze FACS data

Figure S2 Dynamics of CD8^{+} T_{CM} cells after *Pb*RAS vaccination protocols

Figure S3 Percentage of CD8^{+} $T_{E/EM}$ cells after *Pb*RAS vaccination protocols

Figure S4 Dynamics of antigen-specific CD8^{+} $T_{E/EM}$ cells

Table S3 Predicted contribution of local hepatic reactivation to T_{RM} levels

Appendix A1 Boost-effect model for interaction between spleen and liver

Appendix A2 Mathematical analysis of CD8^{+} T cell differentiation dynamics

Appendix A3 Mathematical analysis of dose-dependencies

Table S1: Experimental Groups

group ID	day of harvest (day post prime)	vaccination scheme (days of immunization)	Exp1 (n)	Exp2 (n)	Challenge (n)
**Normal dose** (1×10^4 *Pb*RAS)					
N1	14	0	3	3	-
N2	28	0-14	3	3	6
	42	0-14	3	3	-
	118	0-14	3	3	7
N3	42	0-14-28	3	3	-
	56	0-14-28	3	-	-
	132	0-14-28	3	-	-
	139	0-14-35	3	-	-
**High dose** (1×10^5 *Pb*RAS)					
H1	14	0	3	-	-
H2	28	0-14	3	3	7
	118	0-14	3	3	8
H3	42	0-14-28	3	-	-
**Sub dose** (1×10^3 *Pb*RAS)					
S1	14	0	3	-	-
S2	28	0-14	3	-	3
S3	42	0-14-28	3	-	3
**Mock control** (SG-debris in equal amount to chosen dose)					
MN1	14	0	3	-	-
MN2	28	0-14	3	-	-
MN3	42	0-14-28	3	-	-
MH2	118	0-14	3	2	-
**Naïve**					
Naïve	28	-	-	-	3
	118	-	-	-	3

Table S1: Different groups of mice considered in the analyses: The table shows the number of animals analyzed within each individual group of mice according to the immunization and challenge schedules shown in Figure 1 and Figure 4A . The group ID consisting of the individual dose and the number of injections, as well as the individual vaccination schemes and days of harvest are indicated. For most of the considered vaccination schedules, two independent experimental runs (Exp1 and Exp2) with 3 animals per group were performed.

Table S2: Protection of mice against challenge with *Pb*ANKA sporozoites

**group** ID	**vaccination scheme** (days of immunization)	**day of challenge** (day post prime)	**no. protected/ no. challenged**	**% protection**
**Short-term protection**[a]				
N2	0-14	28	3/6	50
H2	0-14	28	7/7	100
S2	0-14	28	0/3	0
S3	0-14-28	42	0/3	0
Naïve	-	28	0/3	0
**Long-term protection**[a]				
N2	0-14	118	5/8	62.5
H2	0-14	118	7/8	87.7
Naïve	-	118	0/3	0
**Long-term protection - re-challenge**[b]				
N2	0-14	139	5/5	100
H2	0-14	139	7/7	100
Naïve	-	139	0/2	0

Table S2: Protection of mice against challenge with *Pb*ANKA sporozoites: The table shows the number and percentage of animals that were protected within each individual group of mice after challenge with *Pb*ANKA sporozoites. Groups were immunized and challenged according to the indicated protocols with the group ID consisting of the individual dose and the number of injections (see also Figure 1 and Figure 4A). Mice were challenged by i.v. injection of 1×10^3 (a) sporozoites. Mice that were protected following long-term challenge (day 118) were re-challenged by i.v. injection of 1×10^4 sporozoites (b) three weeks after the previous challenge. Protection was defined as the absence of blood-stage parasites.

Figure S1: Gating strategy used to analyze FACS data.

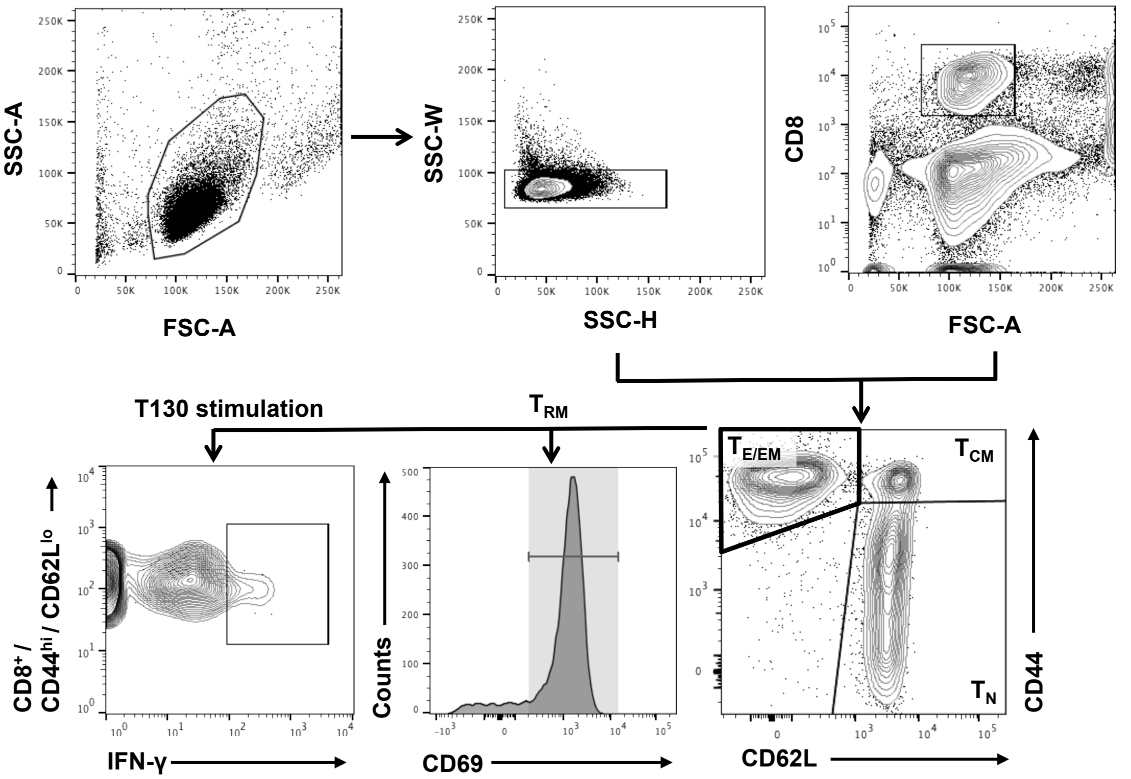

Figure S1: Gating strategy used to analyze FACS data. First, lymphocytes were gated based on forward-scatter (FSC) and side-scatter (SSC) properties. Following doublet discrimination (SSC-W vs. SSC-H strategy) and combination with $CD8^+$ cells (Boolean AND gate), $T_{E/EM}$ (effector/effector memory), T_{CM} (central memory) and T_N (naïve) cells were discriminated by CD44 and CD62L expression. $CD8^+/CD44^{hi}/CD62L^{lo}$ cells were further examined for CD69 expression to determine T_{RM} (resident memory) cells or — following stimulation with peptide *Pb*T130 — gated for IFN-γ-positive cells.

Figure S2: Dynamics of CD8$^+$ T$_{\mathrm{CM}}$ cells after varying *Pb*RAS vaccination protocols.

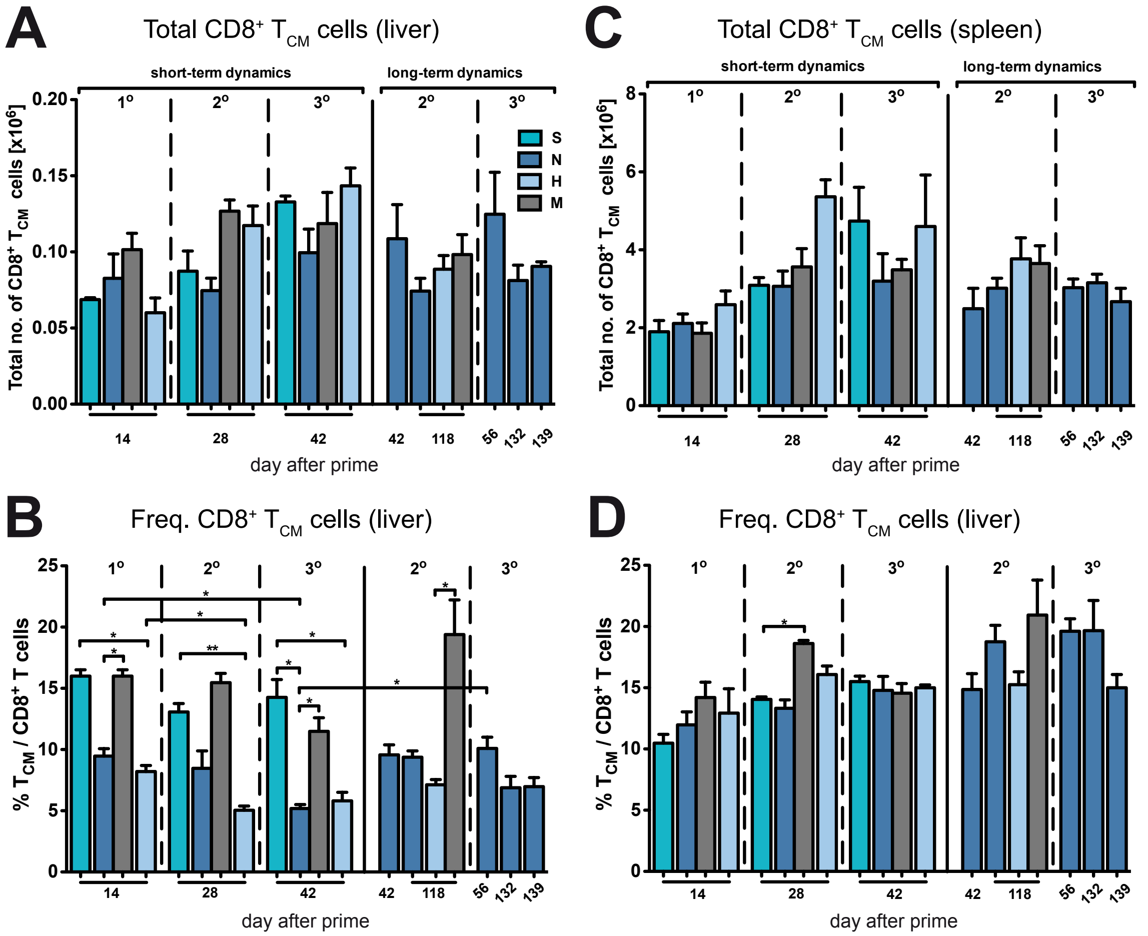

Figure S2: Dynamics of CD8$^+$ T$_{\mathrm{CM}}$ cells after varying *Pb*RAS vaccination protocols. CD8$^+$ T$_{\mathrm{CM}}$ responses were measured in the liver (**A**, **B**) and spleen (**C**, **D**) of mice receiving prime (1°), prime-boost (2°), or prime-boost-boost (3°) immunizations with S-, N-, H-dose or Mock control (M) (compare to Figure 1). The total number of cells per organ (**A**, **B**) or percentages (**C**, **D**) of cells looking at short-term (measurements taken 14 days after last injection) and long-term dynamics (>14 days after last injection) are shown. Numbers below the plots indicate the time point of measurement in days post prime. Numbers of animals per group are specified within Table S1. Graph bars depict means with SEM; *p<0.05; **p<0.01; ***p<0.001; multiple nonparametric rank-based relative comparison.

Figure S3: Percentage of CD8$^+$ T$_{E/EM}$ cells after varying *Pb*RAS vaccination protocols.

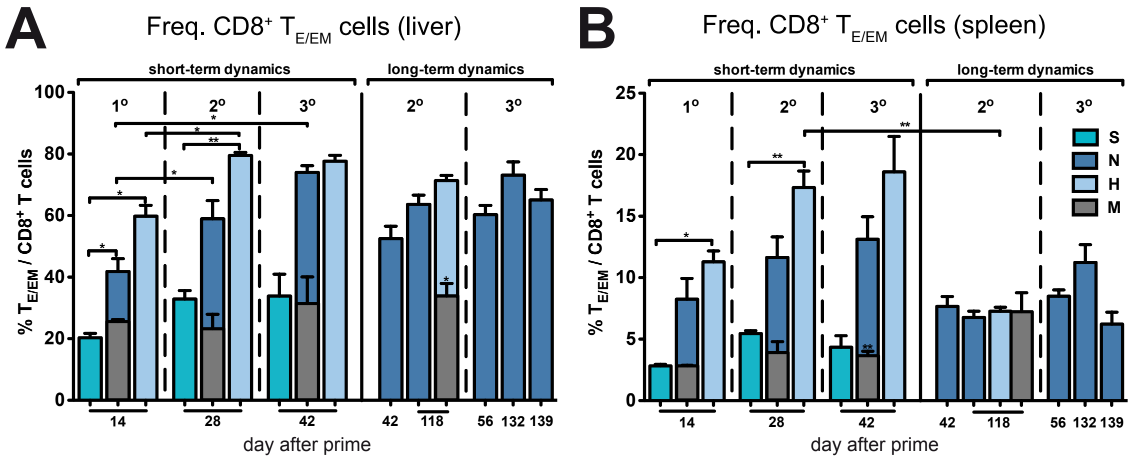

Figure S3: Percentage of CD8$^+$ T$_{E/EM}$ cells after varying *Pb*RAS vaccination protocols. Percentage of CD8$^+$ T$_{E/EM}$ cells was measured in the liver (**A**) and spleen (**B**) of mice receiving prime (1°), prime-boost (2°), or prime-boost-boost (3°) immunizations with S-, N-, H- or MN-dose (compare to Figure 1). Short-term (measurements taken 14 days after last injection) and long-term dynamics (>14 days after last injection) are shown. Numbers below the plots indicate the time point of measurement in days post prime. Numbers of animals per group are specified within Table S1. Graph bars depict means with SEM; *p<0.05; **p<0.01; ***p<0.001; multiple nonparametric rank-based relative comparison.

Figure S4: Dynamics of antigen-specific CD8$^+$ T$_{\mathrm{E/EM}}$ cells after varying *Pb*RAS vaccination protocols.

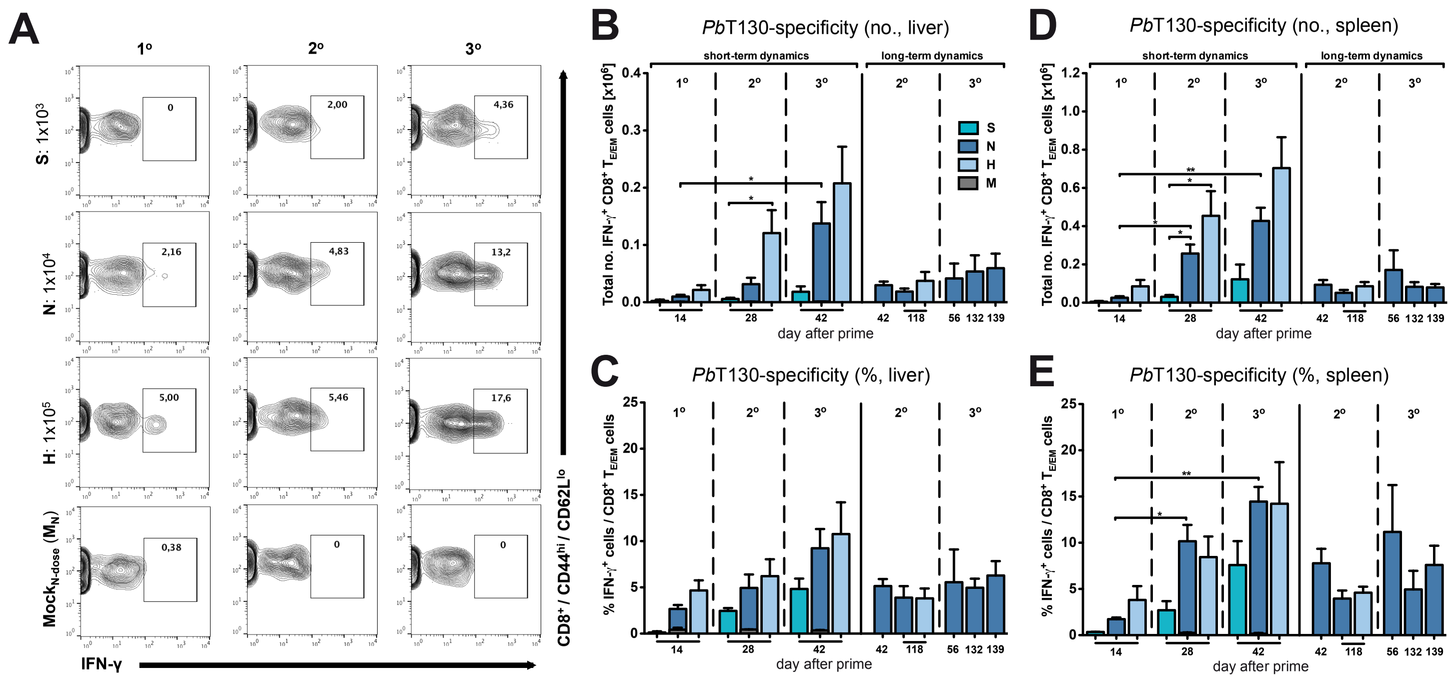

Figure S4: Dynamics of antigen-specific CD8$^+$ T$_{\mathrm{E/EM}}$ cells after varying *Pb*RAS vaccination protocols. Antigen-specificity was measured by IFN-γ expression of CD8$^+$ T$_{\mathrm{E/EM}}$ cells following overnight-stimulation with *Pb*T130 (see *Materials and Methods*). (**A**) Representative FACS-plots of antigen-specific CD8$^+$ T$_{\mathrm{E/EM}}$ cell responses measured in the liver of mice receiving prime (1°), prime-boost (2°), or prime-boost-boost (3°) immunizations with S-, N-, H- or MN-dose (compare to Figure 1). *Pb*T130-specific responses were analyzed in the liver (**B**, **C**) and spleen (**D**, **E**). Shown are total numbers per organ (**B**, **D**) or percentages (**C**, **E**) of cells looking at short-term (measurements taken 14 days after last injection) and long-term dynamics (>14 days after last injection). Numbers below the plots indicate the time point of measurement in days post prime. Numbers of animals per group are specified within Table S1. Graph bars depict means with SEM; *p $<$ 0.05; **p $<$ 0.01; ***p $<$ 0.001; multiple nonparametric rank-based relative comparison.

Table S3: Predicted contribution of local hepatic reactivation to T_{RM} -levels

dose	prime	1st boost	2nd boost
**Proportion of T_{RM} proliferation in novel response**			
**N-dose**	0.28 (± 0.025)	0.84 (± 0.077)	0.92 (± 0.038)
**H-dose**	0.30 (± 0.028)	0.86 (± 0.070)	0.94 (± 0.030)
**S-dose**	0.24 (± 0.018)	0.84 (± 0.105)	0.86 (± 0.086)
**Proportion of T_{RM} proliferation in total response**			
**N-dose**	0.28 (± 0.025)	0.62 (± 0.036)	0.77 (± 0.034)
**H-dose**	0.30 (± 0.028)	0.65 (± 0.037)	0.81 (± 0.032)
**S-dose**	0.24 (± 0.018)	0.50 (± 0.028)	0.61 (± 0.034)

Table S3: Contribution of T_{RM} proliferation during priming and boosting: Estimated proportion of hepatic $CD8^+$ T cell responses that originate from local T_{RM} proliferation based on the mathematical model distinguishing between newly generated cells after each injection and the total response. The remaining proportion (1-x) is due to infiltration from the spleen (see also Figure 6C). Numbers in brackets indicate the standard deviation over 23 independent model predictions with peak-levels of $T_{E/EM}$ cells in the liver below 5×10^7 cells. The analysis indicates that booster injections mainly reactivate local T_{RM} populations.

Appendix A1: A mathematical boost-effect model for describing the interaction between spleen and liver

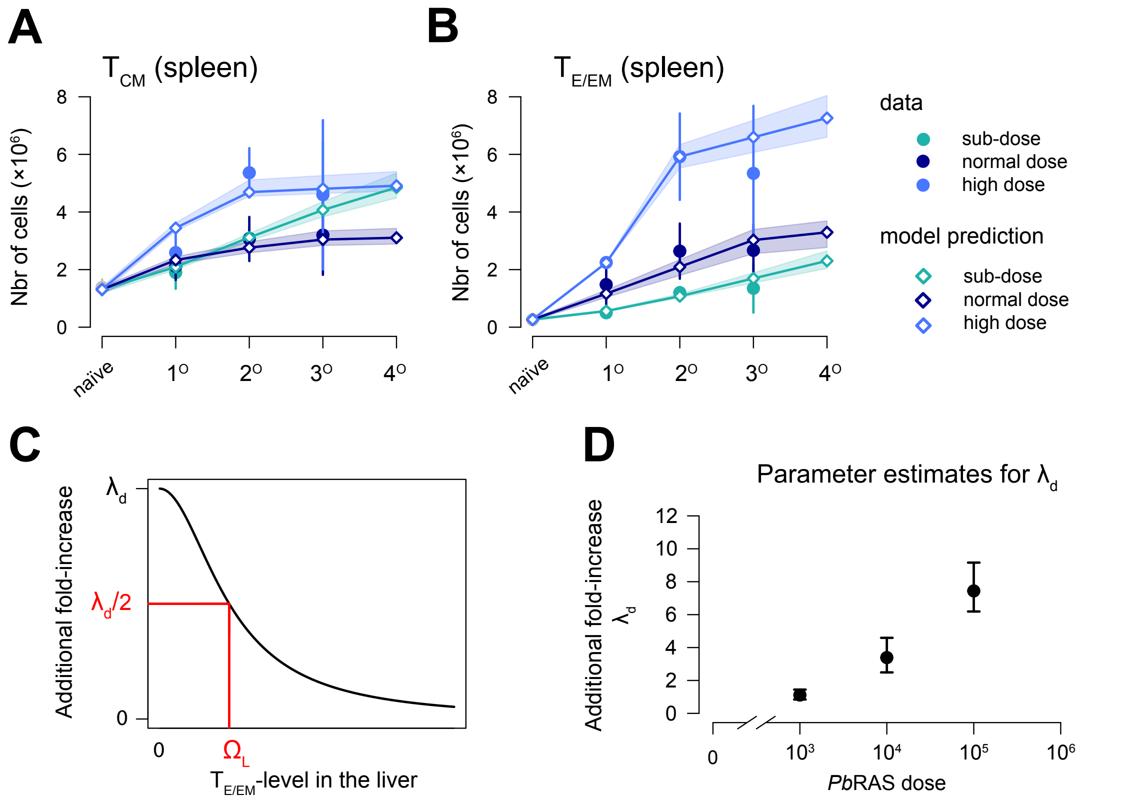

Figure A1.1: Predicted booster effects assuming dependence of splenic activation on $\mathrm{T_{E/EM}}$ cell levels in the liver. Predicted booster effects on the number of $\mathrm{T_{CM}}$ (**A**) and $\mathrm{T_{E/EM}}$ cells (**B**) in the spleen. The plots show the measured data (mean (filled circles) + SEM) for the sub-protective (cyan), normal (darkblue) and high dose (lightblue) for different numbers of injections. Measurements were always taken 14 days after the last injection. White diamonds indicate the corresponding model predictions of the best fit using a model assuming that the level of $\mathrm{T_{E/EM}}$ cells in the liver affects the additional increase of cells in the spleen (see *Materials and Methods* for a detailed explanation of the mathematical model). (**C**) The estimated parameter λ_d indicates the additional fold increase dependent on the dose d, with Ω_L determining the level of $\mathrm{T_{E/EM}}$ cells in the liver at which the additional fold increase in the spleen reduces to half of the maximal value. (**D**) Estimates for λ_d dependent on the used dose of *Pb*RAS. The corresponding parameter estimates are shown in **Table A1.1**. Shaded areas in (**A**) and (**B**) indicate the 95%-confidence intervals of model predictions.

**parameter**	$\mathbf{T}_{\mathrm{CM}}$ **spleen**	$\mathbf{T}_{\mathrm{E/EM}}$ **spleen**
T_0 ($\times 10^6$ cells)	1.31 (1.15, 1.47)	0.27 (0.22, 0.31)
λ_S	0.60 (0.46, 0.75)	1.12 (0.84, 1.45)
λ_N	0.78 (0.50, 1.10)	3.39 (2.51, 4.59)
λ_H	1.63 (1.26, 2.04)	7.44 (6.19, 9.15)
Ω_L ($\times 10^6$ cells)	0.23 (0.18, 0.29)	

Table A1.1: Estimated rates of the maximal dose-dependent increase of $\mathbf{T}_{\mathrm{CM}}$ and $\mathbf{T}_{\mathrm{E/EM}}$ cells in the spleen during prime. Estimates were obtained using a mathematical model assuming that the prior-level of $\mathrm{T}_{\mathrm{E/EM}}$ cells in the liver affects additional cell growth in the spleen. Estimates are obtained using Eq.(2) in *Materials and Methods* with a Hill-coefficient of $\tau = 2$ as it provided slightly improved estimates compared to a Hill-coefficient of $\tau = 1$ (AICc= 26.8 vs. 28.0). Values indicate the best fit with numbers in brackets representing 95%-confidence intervals that were obtained by profile-likelihood analysis. The parameter λ_d is defined as the additional fold-increase per boost, i.e., $1 + \lambda_d$ defines the fold-change in absolute cell numbers.

Appendix A2: Mathematical analysis of CD8$^+$ T cell differentiation dynamics

A2.1 A mathematical model to describe CD8$^+$ T cell subset dynamics in spleen and liver

The mathematical model considers the dynamics of five different CD8$^+$ T cell subsets: naïve (T_{N}), central memory (T_{CM}), and effector/effector memory in spleen ($T_{\mathrm{E/EM}}$ in spleen, in the following abbreviated by T_{EMS}), as well as effector/effector memory cells in the liver, which are divided into circulating effector/ effector memory cells (T_{EML}) and tissue-resident (T_{RM}) cells in the liver. Each cellular subset (except for naïve cells) is assumed to have a cellular turnover according to individual net-proliferation rates $\rho_{\cdot}$. Cell differentiation and migration between different subsets occurs according to corresponding rates indicated by the parameter $\sigma_{\cdot}$. All possible differentiation and proliferation dynamics considered are depicted in **Figure A2.1**.

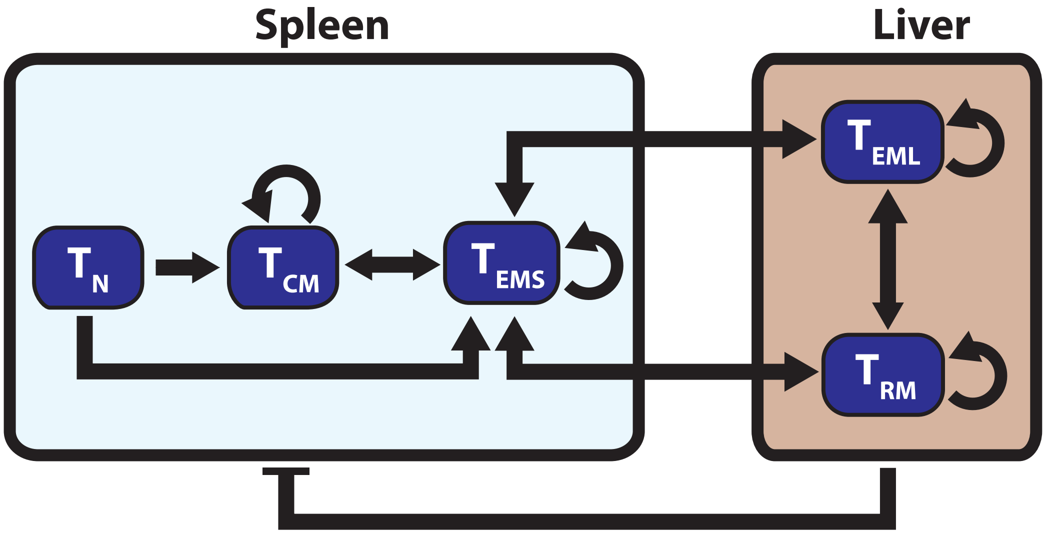

Figure A2.1: The general model of CD8$^+$ T cell turnover: The general model takes into account all possible interactions between subsets and organs with respect to net-proliferation and cell differentiation.

We assume that each prime or booster injection at time point T_i, $i \geq 1$ leads to a specified duration of antigen presence L_i, $i \geq 1$ during which parameters might change their value. Thus, each model parameter (except for the naïve cell differentiation) consists of an antigen-dependent and

an antigen-independent, i.e. baseline, part. The presence of antigen is modelled by an indicator function, which leads to an increase in the parameter values for the respective duration of antigen presence. Therefore, the structure of model parameters is of type

$$\alpha := \mathbb{1}_{\{t \in T_{\mathrm{ag}}\}} \alpha^{\mathrm{ag}} + \alpha^{\mathrm{b}} \ , \tag{1}$$

with α^{ag} and $\alpha^{\mathrm{b}} \in \mathbb{R}$ denoting the antigen-dependent and baseline rates, respectively. The set T_{ag} contains the intervals in which antigen is present to stimulate the cellular turnover and is defined as $T_{\mathrm{ag}} := \bigcup_{i=1}^{n} [T_i, T_i + L_i]$, where $n \in \mathbb{N}$ defines the total number of shots.

Based on our previous analysis (**Appendix A1**), we assume that antigen-dependent proliferation of central memory cells in the spleen is reduced the more T_{RM} cells are present in the liver. This negative feedback is modelled by a Hill-equation and is defined as

$$\gamma(\mathrm{RM}) := 1 - \frac{T_{\mathrm{RM}}^2}{T_{\mathrm{RM}}^2 + \Omega_{\mathrm{RM}}^2} \ . \tag{2}$$

Based on the results of **Appendix A1** we set $\Omega_{\mathrm{RM}} = 2.36 \times 10^5$ cells.

In summary, the global model comprising all possible CD8$^+$ T cell interaction dynamics is described by the following system of ordinary differential equations:

$$\begin{aligned}
\frac{dT_{\mathrm{N}}}{dt} &= -\mathbb{1}_{\{t \in T_{\mathrm{AG}}\}} \left(\sigma^{\mathrm{ag}}_{\mathrm{N-CM}} + \sigma^{\mathrm{ag}}_{\mathrm{N-EMS}} \right) T_{\mathrm{N}} \\
\frac{dT_{\mathrm{CM}}}{dt} &= \mathbb{1}_{\{t \in T_{\mathrm{AG}}\}} \left(\sigma^{\mathrm{ag}}_{\mathrm{N-CM}} T_{\mathrm{N}} + \gamma(\mathrm{RM}) \rho^{\mathrm{ag}}_{\mathrm{CM}} T_{\mathrm{CM}} \right) + (\rho^{\mathrm{b}}_{\mathrm{CM}} - \sigma_{\mathrm{CM-EMS}}) T_{\mathrm{CM}} \\
&\quad + \sigma_{\mathrm{EMS-CM}} T_{\mathrm{EMS}} \\
\frac{dT_{\mathrm{EMS}}}{dt} &= -\mathbb{1}_{\{t \in T_{\mathrm{AG}}\}} \sigma^{\mathrm{ag}}_{\mathrm{N-EMS}} T_{\mathrm{N}} + \sigma_{\mathrm{CM-EMS}} T_{\mathrm{CM}} \\
&\quad + \left(\rho_{\mathrm{EMS}} - \sigma_{\mathrm{EMS-EML}} - \sigma_{\mathrm{EMS-RM}} \right) T_{\mathrm{EMS}} \\
&\quad + \sigma_{\mathrm{EML-EMS}} T_{\mathrm{EML}} + \sigma_{\mathrm{RM-EMS}} T_{\mathrm{RM}} \\
\frac{dT_{\mathrm{EML}}}{dt} &= \sigma_{\mathrm{EMS-EML}} T_{\mathrm{EMS}} + \left(\rho_{\mathrm{EML}} - \sigma_{\mathrm{EML-EMS}} - \sigma_{\mathrm{EML-RM}} \right) T_{\mathrm{EML}} + \sigma_{\mathrm{RM-EML}} T_{\mathrm{RM}} \\
\frac{dT_{\mathrm{RM}}}{dt} &= \sigma_{\mathrm{EMS-RM}} T_{\mathrm{EMS}} + \sigma_{\mathrm{EML-RM}} T_{\mathrm{EML}} + \left(\rho_{\mathrm{RM}} - \sigma_{\mathrm{RM-EMS}} - \sigma_{\mathrm{RM-EML}} \right) T_{\mathrm{RM}}
\end{aligned} \tag{3}$$

The global model contains 26 unknown proliferation and differentiation parameters. Additionally the duration of antigen presence after the second, L_2 and third shot, L_3 are also unknown, meaning a total of 28 parameters needs to be estimated for the global model. The duration of antigen presence after prime was fixed to $L_1 = 8$.

A2.2 Unbiased model selection algorithm

The aim of the unbiased model selection algorithm is to find the most appropriate submodel of the global model (Eq. (3) and **Figure A2.1**) for describing the experimental data. Based on a specific submodel, the algorithm tests all neighboring models, which are calculated based on the current selected model, and updates the current model if a better one is found. Therefore, the algorithm successively progresses through the space of all possible models and stops if no better model can be found. The details of the unbiased model selection algorithm are explained below.

Critical parameter sets To avoid the testing of models that are not able to reproduce the cellular dynamics we identified 6 critical parameter sets:

- $\mathrm{S}_1 = \{\sigma^{\mathrm{ag}}_{\mathrm{N-CM}}; \sigma^{\mathrm{ag}}_{\mathrm{EMS-CM}}; \sigma^{\mathrm{b}}_{\mathrm{EMS-CM}}\}$ (differentiation into $\mathrm{T_{CM}}$)
- $\mathrm{S}_2 = \{\sigma^{\mathrm{ag}}_{\mathrm{N-EMS}}; \sigma^{\mathrm{ag}}_{\mathrm{CM-EMS}}; \sigma^{\mathrm{b}}_{\mathrm{CM-EMS}}\}$ (differentiation into $\mathrm{T_{EMS}}$)
- $\mathrm{S}_3 = \{\sigma^{\mathrm{ag}}_{\mathrm{EMS-EML}}; \sigma^{\mathrm{b}}_{\mathrm{EMS-EML}}; \sigma^{\mathrm{ag}}_{\mathrm{RM-EML}}\}$ (differentiation into $\mathrm{T_{EML}}$)
- $\mathrm{S}_4 = \{\sigma^{\mathrm{ag}}_{\mathrm{EMS-RM}}; \sigma^{\mathrm{b}}_{\mathrm{EMS-RM}}; \sigma^{\mathrm{ag}}_{\mathrm{EML-RM}}; \sigma^{\mathrm{b}}_{\mathrm{EML-RM}}\}$ (differentiation into $\mathrm{T_{RM}}$)
- $\mathrm{S}_5 = \{\rho^{\mathrm{ag}}_{\mathrm{EMS}}\}$ (antigen-dependent proliferation of $\mathrm{T_{EMS}}$)
- $\mathrm{S}_6 = \{\rho^{\mathrm{ag}}_{\mathrm{EML}}\}$ (antigen-dependent proliferation of $\mathrm{T_{EML}}$)

Each model has to contain at least one parameter of each critical set in order to be tested. If this condition is not fulfilled the respective model is discarded.

The outline of the algorithm Starting from an initial model, the unbiased model selection algorithm is searching for superior models explaining the experimental data based on the combination of three different methods:

- **Forward search:** Adds one parameter to the current model
- **Double forward search:** Adds two parameters to the current model
- **Backward elimination:** Removes one of the current model parameters

At each step, the algorithm generates a new set of models based on the chosen method and tests their performance in comparison to the current model. If the current model contains k (out of n possible) different parameters, the algorithm will generate k new models for a backward elimination and $n-k$ models for a forward search. All models that do not violate the critical condition specified above are used for testing. Each model is fitted multiple times to the data using the `optim`-fitting routine in `R` with 5 different starting conditions for the parameter values (including the parameter set of the current model). The estimated parameter values of the best fit and the corresponding

AICc value is returned. The current model is updated if one of the models shows better performance according to the AICc. The different methods (forward search, double-forward search and backward elimination) are used in successive steps as described below. If none of the different methods leads to a better model, the algorithm is halted and the best model is returned.

In summary, the exact testing sequence of the algorithm is given as follows:

- Read in the initial model. Jump to the initially specified testing method.
- Forward search
  1. For each non-model parameter, create a new model by adding the parameter to the current model. Test all models.
  2. If a better model is found, update the current model and continue with forward search
  3. If no better model is found:
     - Continue with backward elimination if the previous run was a forward or a double forward search
     - Continue with double forward search if the previous run was a backward elimination
- Double forward search
  1. For each combination of two non-model parameters, create a new model by adding the two parameters to the current model. Test all models.
  2. If a better model is found, update the current model and continue with forward search
  3. If no better model is found, halt the simulation and return the currently best model and estimated parameter values
- Backward elimination
  1. For each model parameter, create a new model by removing the parameter from the current model. Test all models that don't violate a critical condition.
  2. If a better model is found, update the current model and continue with backward elimination
  3. If no better model is found:
     - Continue with forward search if the previous run was a backward elimination
     - Continue with double forward search if the previous run was a forward search

Selected starting models: In total, we applied our unbiased model selection algorithm to ten different models. These models included (a) the global model, (b) 3 minimal models, in which we

selected one parameter from each critical set of parameters, and (c) 6 different models, in which we selected one parameter from each critical set of parameters and added randomly five of the remaining parameters. This would mean, that the models at the start comprised 26 unknown parameters in (a), 8 (6 + 2 for L_2 and L_3) unknown parameters in (b) and 13 (11 + 2 for L_2 and L_3) unknown parameters in (c). Each starting model in (b) and (c) was selected at random.

A2.3 Parameter identifiability and model evaluation

The unbiased model selection algorithm allows the identification of systematic relationships between the different cellular subsets, as well as quantification of the corresponding differentiation and proliferation rates. However, due to model complexity, some of the parameters, especially those characterizing the expansion phases in response to immunizations, turn out to be non-identifiable due to limited data availability. **Figure A2.2** shows the profile likelihood analysis of the estimated parameters for the selected differentiation model (Figure 6B). These profiles indicate practical non-identifiability for some of the parameters, e.g. $\sigma^{\mathrm{ag}}_{\mathrm{CM-EMS}}$ and $\rho^{\mathrm{ag}}_{\mathrm{EML}}$ [1].

To analyze the support for and the robustness of the selected model in comparison to all other differentiation models tested, we additionally determined the Akaike weights for each model [2]. Calculating the evidence ratio based on the Akaike weights we determined how much more likely a model that includes a certain parameter is in describing the data than a model that does not include this parameter (**Table A2.2**). The evidence ratio indicates a high evidence for the selected model shown in Figure 6B in the main manuscript.

References

[1] Raue A, Kreutz C, Maiwald T, Bachmann J, Schilling M, Klingmuller U, et al. Structural and practical identifiability analysis of partially observed dynamical models by exploiting the profile likelihood. Bioinformatics. 2009 Aug;25(15):1923–1929.

[2] Burnham KP, Anderson DR. Model selection and multimodel inference. 2nd ed. New York ; Berlin ; Heidelberg [u.a.]: Springer; 2003. S. 455 - 484.

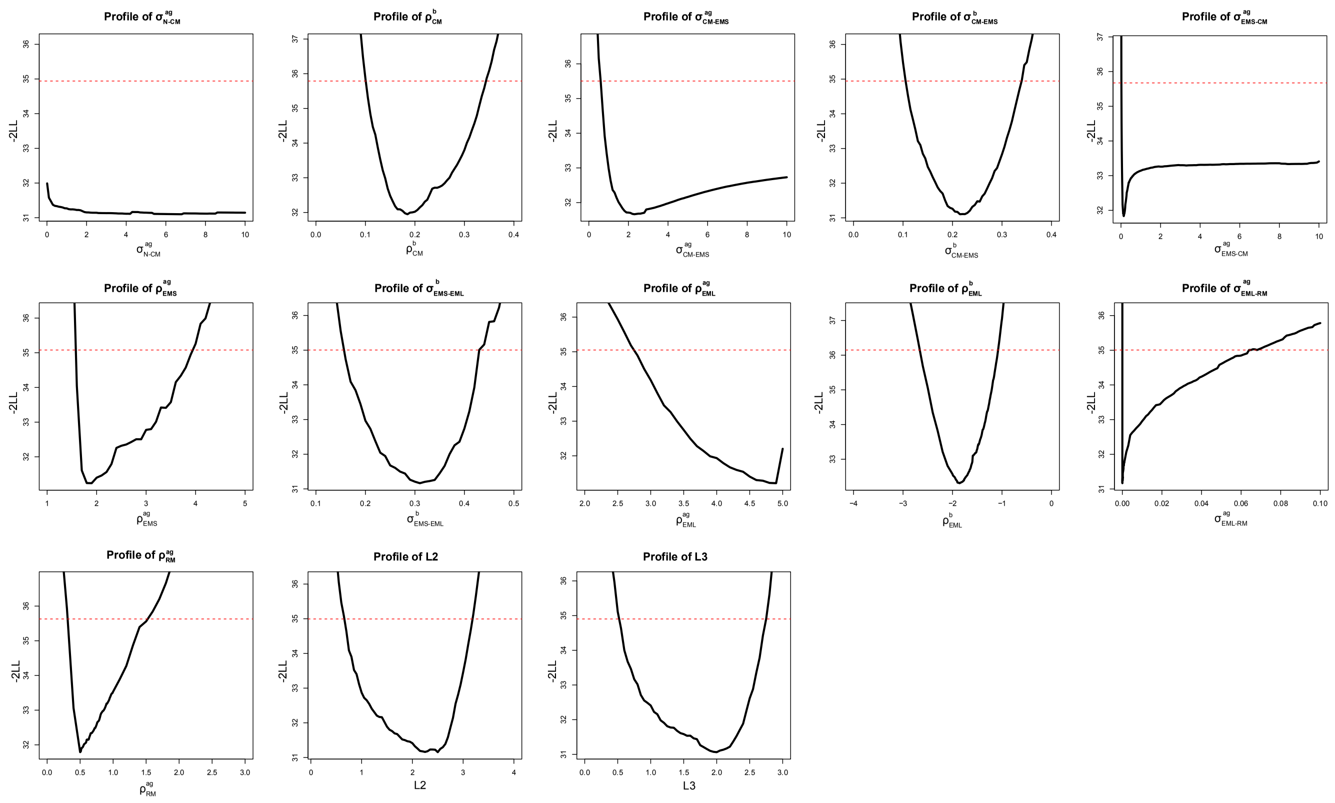

Figure A2.2: The profile likelihoods of the estimated model parameters given the selected model (Figure 6B). All parameter values with a negative log-likelihood value (-2LL) below the red dotted line (minimum value +3.84) are within the considered 95% confidence interval of the parameter. Several parameters indicate practical non-identifiability due to insufficient data availability for the expansion phases.

parameter	evidence ratio	normalised probability
σ^{ag}_{N-CM}	44.9	0.978
σ^{ag}_{N-EMS}	9×10^{-2}	0.083
ρ^{ag}_{CM}	6.8×10^{-2}	0.064
ρ^{b}_{CM}	3.1×10^{4}	0.999
σ^{ag}_{CM-EMS}	1.7×10^{4}	0.999
σ^{b}_{CM-EMS}	7×10^{4}	0.999
σ^{ag}_{EMS-CM}	3.9×10^{4}	0.999
σ^{b}_{EMS-CM}	3.9×10^{-2}	0.038
ρ^{ag}_{EMS}	always included	1
ρ^{b}_{EMS}	1.1×10^{-1}	0.102
$\sigma^{ag}_{EMS-EML}$	4.7×10^{-2}	0.046
$\sigma^{b}_{EMS-EML}$	4.1×10^{4}	0.999
$\sigma^{ag}_{EML-EMS}$	4.2×10^{-2}	0.04
$\sigma^{b}_{EML-EMS}$	4.1×10^{-2}	0.039
ρ^{ag}_{EML}	always included	1
ρ^{b}_{EML}	2.1×10^{4}	0.999
σ^{ag}_{EML-RM}	44.9	0.978
σ^{b}_{EML-RM}	4×10^{-2}	0.039
σ^{ag}_{RM-EML}	4.2×10^{-2}	0.041
ρ^{ag}_{RM}	15.2	0.938
ρ^{b}_{RM}	8.8×10^{-2}	0.082
σ^{ag}_{EMS-RM}	6.6×10^{-2}	0.062
σ^{b}_{EMS-RM}	4.2×10^{-2}	0.041
σ^{ag}_{RM-EMS}	4.6×10^{-2}	0.044
L_2	always included	1
L_3	always included	1

Table A2.2: The evidence ratios based on the Akaike weights show how much more likely a model is that includes the respective parameter. The normalised probability shows the fraction that the Akaike weights of the models, which include the respective parameter, have compared to all weights. Akaike weights are calculated based on the 500 best models. Blue colour indicates the parameters of the best fitting model.

Appendix A3: Modelling dose-dependency

To determine the effect of different immunization doses on the $CD8^{+}$ T cell differentiation dynamics, we used the differentiation model that was identified by analyzing the N-dose vaccinated mice (Figure 6B) and incorporated a dose-dependent factor, d. This factor increases or decreases the corresponding antigen-dependent proliferation and differentiation rates dependent on the dose analyzed, with the normal dose defining the baseline ($d = 1$).

We fitted all possible combinations for applying the dose factor d to the antigen-dependent rates, leading to 511 different models for each dose, i.e., H- and S-dose. For the analysis, all parameters were fixed to the values obtained for the N-dose immunization scenario and only the dose-dependent factor d was estimated. To account for limited parameter identifiability due to model complexity and validate the robustness of the analysis, we used ten different parameter sets that were estimated for the N-dose (**Table A3.1**).

Again, models were ranked based on their ability to explain the experimental data using the corrected Akaike information criterion. **Table A3.2** and **Table A3.3** show the best performing models for the H- and S-dose, respectively for each of the ten different parameter sets given in Table A3.1. The results indicate that the changed dynamics for high- and sub-dose vaccinated animals can best be explained by dose-dependent changes to the differentiation rate from T_{CM} to $T_{E/EM}$ in the spleen, σ^{ag}_{CM-EMS}, and a changed net-proliferation rate of T_{RM} in the liver, ρ^{ag}_{RM}. While a 10-fold higher vaccination dose (H-dose) leads to a 1.2-fold increase in these rates compared to the normal dose of 1×10^4 *Pb*RAS, immunization by a 10-fold lower, sub-protective dose (S-dose) leads to a reduction of around 80% of their baseline value. Corresponding model predictions of the estimated dynamics for each cellular subset are shown in **Figure A3.1** and **Figure A3.2**.

Figure A3.3 shows the comparison of the different dosage effects as predicted by the mathematical model (Figure 6B). As can be seen in the plots, H- and N-dose immunizations do not lead to increased $T_{E/EM}$ cell levels in the spleen after more than one booster injection. Increasing the number of immunizations with the subprotective dose, our model would also predict an increase in the T_{RM} cells in the liver (**Figure A3.4**). However, 11 different injections in 14 day intervals would be necessary to reach similar T_{RM} cell levels than mice receiving three N-dose vaccinations with reaching levels that are obtained by three immunizations with the N-dose. Nevertheless, here our model would also predict unrealistic dynamics for total $T_{E/EM}$ cell levels in the spleen and liver, indicating that additional factors, such as T cell exhaustion due to subsequent activation, should be considered in order to predict the effect of multiple vaccinations.

parameter	unit	Set 1	Set 2	Set 3	Set 4	Set 5	Set 6	Set 7	Set 8	Set 9	Set 10
-2LL	-	33	33.1	33.3	33.4	33.4	33.6	33.7	34.1	34.3	34.4
T_{EML} peak	cells	4×10^{7}	4.2×10^{7}	3.2×10^{7}	3.3×10^{7}	3×10^{7}	2.9×10^{7}	2.1×10^{7}	1.5×10^{7}	4.3×10^{7}	9.2×10^{6}
σ^{ag}_{N-CM}	day^{-1}	2.9×10^{-5}	2.2×10^{-1}	2.2×10^{-1}	5.6×10^{-6}	1.5×10^{-5}	2.8×10^{-6}	1.4×10^{-1}	1.2×10^{-1}	1.4×10^{-6}	1.1×10^{-1}
ρ^{b}_{CM}	day^{-1}	0.16	0.14	0.14	0.14	0.17	0.14	0.13	0.13	0.12	0.13
σ^{ag}_{CM-EMS}	day^{-1}	3.74	3.02	3.11	5.99	4.1	5.21	3.32	3.59	6.47	3.52
σ^{b}_{CM-EMS}	day^{-1}	0.16	0.14	0.14	0.14	0.17	0.14	0.13	0.13	0.12	0.13
σ^{ag}_{EMS-CM}	day^{-1}	0.25	0.27	0.28	0.45	0.24	0.44	0.32	0.37	0.7	0.37
ρ^{ag}_{EMS}	day^{-1}	3.2	1.95	1.95	3.4	3.3	3.5	2	2	3.6	2.03
$\sigma^{b}_{EMS-EML}$	day^{-1}	0.23	0.2	0.2	0.2	0.24	0.2	0.19	0.18	0.17	0.18
ρ^{ag}_{EML}	day^{-1}	4.99	3.7	3.6	4.99	5	4.99	3.5	3.4	4.99	3.3
ρ^{b}_{EML}	day^{-1}	-1.8	-1.62	-1.57	-1.61	-1.79	-1.53	-1.5	-1.4	-1.35	-1.43
σ^{ag}_{EML-RM}	day^{-1}	8.5×10^{-3}	5.1×10^{-3}	6.3×10^{-3}	1×10^{-2}	1×10^{-2}	1.2×10^{-2}	9.4×10^{-3}	1.2×10^{-2}	7.8×10^{-3}	1.8×10^{-2}
ρ^{ag}_{RM}	day^{-1}	0.74	0.53	0.53	0.92	0.74	0.96	0.56	0.59	1.19	0.59
L_2	days	1.06	1.52	1.5	0.89	1.04	0.84	1.42	1.36	0.72	1.34
L_3	days	0.86	1.2	1.18	0.7	0.86	0.67	1.12	1.06	0.55	1.04

Table A3.1: Fixed parameter sets estimated for the N-dose vaccination protocol and used for dose fitting: The ten best parameter sets that were estimated for the selected differentiation model (see Figure 6B) analyzing the N-dose immunization, and that generated $T_{E/EM}$ cell numbers below the threshold of 5×10^7 cells at the peak. The model performance indicated by the negative log-likelihood value (-2LL) and the individual rate constants are shown. As could already be seen in **Figure A2.2**, antigen-dependent differentiation and proliferation rates show generally larger variations than baseline rates.

**-2LL**	**dose factor**, d	σ^{ag}_{N-CM}	σ^{ag}_{CM-EMS}	σ^{ag}_{EML-RM}	ρ^{ag}_{RM}	**L2**
50.0	1.19	X	X	-	X	X
50.3	1.19	X	X	-	X	X
50.7	1.23	-	X	-	X	X
51.8	1.17	X	X	X	X	X
52.2	1.16	X	X	X	X	X
52.2	1.16	X	X	X	X	X
52.9	1.17	X	X	X	X	X
53.8	1.16	X	X	X	X	X
54.3	1.15	X	X	X	X	X
55.0	1.18	X	X	-	X	X

Table A3.2: Identified dose-dependent parameters for H-dose vaccination: For each of the ten best parameter sets given in **Table A3.1**, 511 different models were tested to determine the dose-dependency. Each row shows the corresponding best fit in terms of the estimated dose factor, the affected differentiation and proliferation rates, and the model performance indicated by the negative log-likelihood (-2LL).

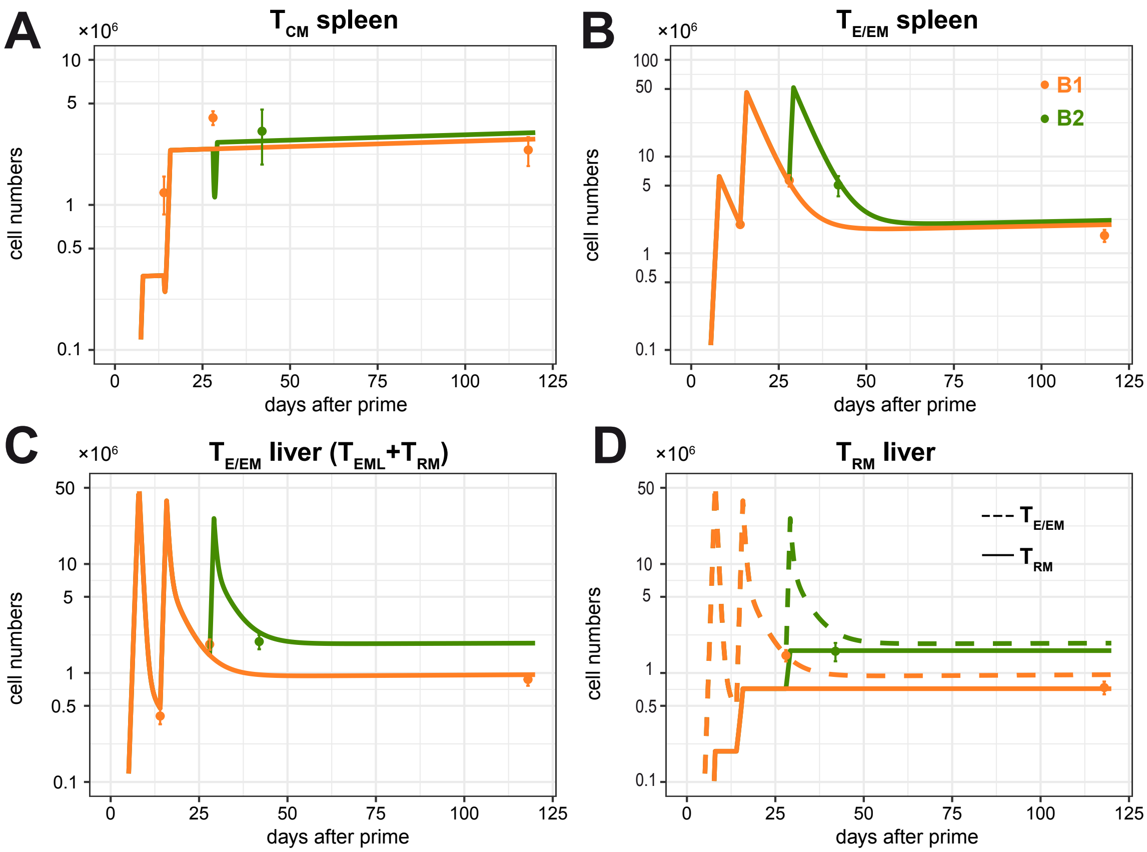

Figure A3.1: Model predictions for H-dose vaccination protocol: Panels show the cellular dynamics of T_{CM} (**A**) and $T_{E/EM}$ (**B**) cells (T_{EMS}) in the spleen, as well as the dynamics for the $T_{E/EM}$ cells (T_{EML} + T_{RM} , **C**) and T_{RM} cells (**D**) in the liver. Lines represent model predictions based on a prime-boost (2°=*B1*, orange) or a prime-boost-boost (3°=*B2*, green) vaccination strategy using a high dose of 1×10^5 *Pb*RAS. The corresponding data are shown as mean ± SEM.

**-2LL**	**dose factor**	$\sigma^{\text{ag}}_{\text{CM}-\text{EMS}}$	$\sigma^{\text{ag}}_{\text{EML}-\text{RM}}$	$\rho^{\text{ag}}_{\text{RM}}$
53.5	0.17	X	-	X
53.8	0.18	X	-	X
55.3	0.19	X	-	X
56.4	0.22	X	X	-
56.6	0.22	X	-	X
57.6	0.22	X	X	-
57.9	0.22	X	X	-
58.9	0.30	X	X	-
62.3	0.31	X	X	-
66.9	0.4	X	X	-

Table A3.3: Identified dose-dependent parameters for S-dose vaccination: For each of the ten best parameter sets given in **Table A3.1**, 511 different models were tested to determine the dose-dependency. Each row shows the corresponding best fit in terms of the estimated dose factor, the affected differentiation and proliferation rates, and the model performance indicated by the negative log-likelihood (-2LL).

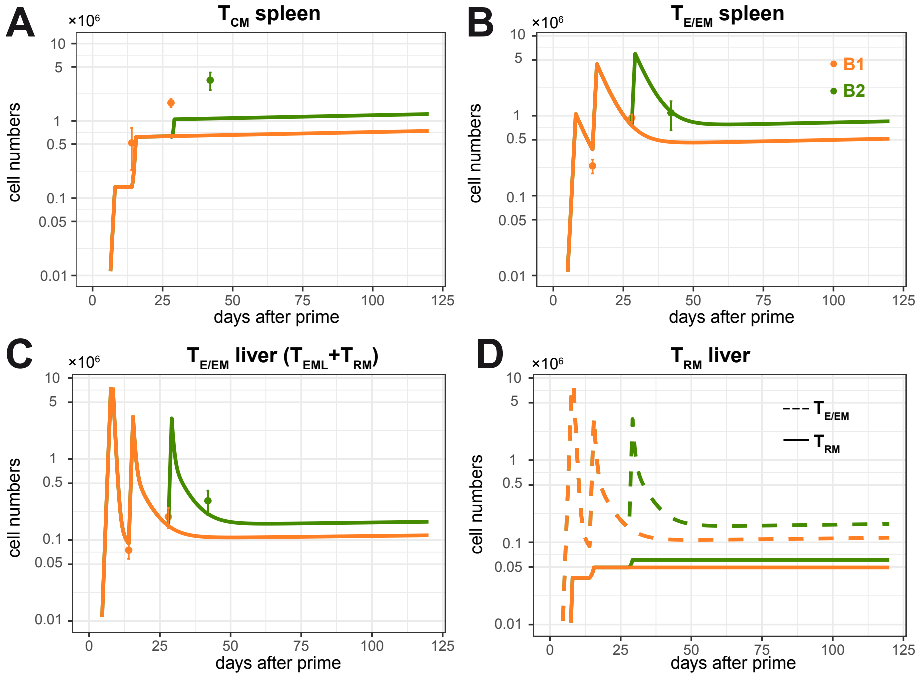

Figure A3.2: Model predictions for S-dose vaccination protocol: Panels show the cellular dynamics of T_{CM} (**A**) and $T_{E/EM}$ (**B**) cells (T_{EMS}) in the spleen, as well as the dynamics for the $T_{E/EM}$ cells (T_{EML} + T_{RM} , **C**) and T_{RM} cells (**D**) in the liver. Lines represent model predictions based on a prime-boost (2°=*B1*, orange) or a prime-boost-boost (3°=*B2*, green) vaccination strategy using a sub-protective dose of 10^3 *Pb*RAS. The corresponding data are shown as mean ± SEM when measured.

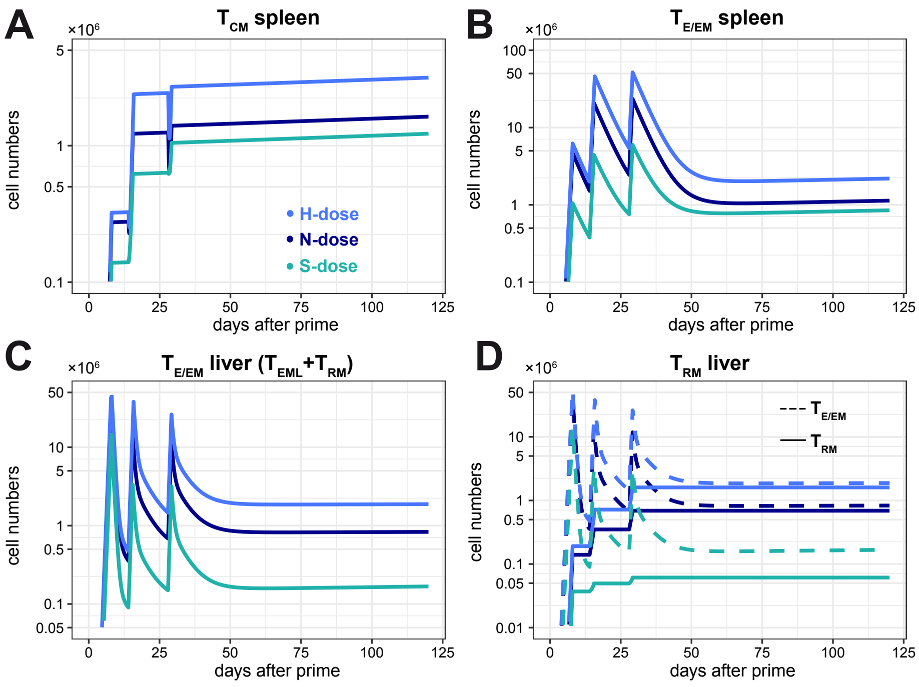

Figure A3.3: Comparing the cellular dynamics of different dosages: Panels show the cellular dynamics of T_{CM} (**A**) and $T_{E/EM}$ (**B**) cells (T_{EMS}) in the spleen, as well as the dynamics for the $T_{E/EM}$ cells (T_{EML} + T_{RM} , **C**) and T_{RM} cells (**D**) in the liver. Lines are based on a prime-boost-boost (3°) vaccination strategy using either a normal (1×10^4 *Pb*RAS - dark blue), high (1×10^5 *Pb*RAS - light blue) or sub-protective dose (1×10^3 *Pb*RAS - turquoise).

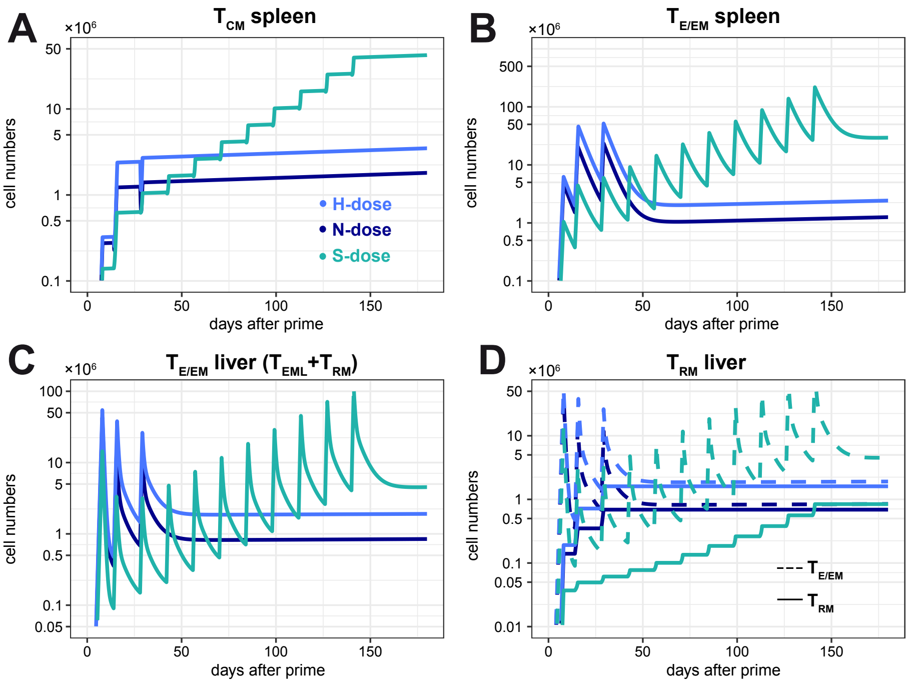

Figure A3.4: Comparing the cellular dynamics of different doses: Panels show the cellular dynamics of T_{CM} (**A**) and $T_{E/EM}$ (**B**) cells (T_{EMS}) in the spleen, as well as the dynamics for the $T_{E/EM}$ cells (T_{EML} + T_{RM} , **C**) and T_{RM} cells (**D**) in the liver. Lines are based on a prime-boost-boost (3°) vaccination strategy using either a normal (1×10^4 *Pb*RAS - dark blue) or high dosage (1×10^5 *Pb*RAS - light blue). For the sub-protective (1×10^3 *Pb*RAS - turquoise) dose, we simulated 11 immunizations in total with a timespan of 14 days between subsequent injections.
